# Exciton hybridization in a WS_2_/MoS_2_ heterobilayer mediated by a surface wave via strong photon–exciton coupling

**DOI:** 10.1515/nanoph-2024-0737

**Published:** 2025-03-14

**Authors:** Junxuan Yi, Shimei Liu, Shulei Li, Weichen He, Zuxin Chen, Sheng Lan

**Affiliations:** Guangdong Provincial Key Laboratory of Nanophotonic Functional Materials and Devices, School of Optoelectronic Science and Engineering, South China Normal University, Guangzhou 510006, China; School of Optoelectronic Engineering, Guangdong Polytechnic Normal University, Guangzhou 510665, China; GBA Branch of Aerospace Information Research Institute, Chinese Academy of Sciences, Guangzhou, 510700, China; Guangdong Provincial Key Laboratory of Terahertz Quantum Electromagnetics, Guangzhou, 510700, China; School of Electronic Science and Engineering (School of Microelectronics), South China Normal University, Foshan, 528225, China

**Keywords:** transition metal dichalcogenide, heterobilayer, surface wave, exciton, strong coupling, hybridization

## Abstract

The hybridization of multiple excitons in a heterobilayer composed of two transition metal dichalcogenides (TMDCs) based on strong light–matter interaction is interesting from the viewpoint of both fundamental research and practical application. Here, we investigate numerically and experimentally the hybridization of three excitons in a heterobilayer mediated by the surface plasmon polaritons (SPPs) excited on a thin Au film and the transverse-electric (TE) polarized waves excited on a Si_3_N_4_/Ag heterostructure via photon–exciton coupling. Relying on numerical simulation, we observe anticrossing behaviors in the angle-resolved reflection spectra calculated for MoS_2_/WS_2_/Au and WS_2_/MoS_2_/Si_3_N_4_/Ag heterostructures, which reveal the coupling between the surface wave (SPPs or TE waves) and the multiple excitons in the heterobilayer. In experiments, we employ the oligomers of polystyrene (PS) nanospheres as scatters to transfer the surface waves into far-field radiations. Similarly, we observe anticrossing behaviors in the angle-resolved scattering spectra measured for the oligomers of PS nanospheres. Relying on the coupled oscillator model, we observe Rabi splitting energies of Ω_SPP_ ∼206.79 meV for the SPPs and Ω_TE_ ∼237.60 meV for the TE waves. Based on the calculated current density distributions and Hopfield coefficients, we demonstrate the hybridization of the three excitons in the WS_2_/MoS_2_ heterobilayer mediated by the TE waves. Our findings open new horizons for manipulating light–matter interaction in TMDC heterobilayers and suggest the potential applications of exciton hybridization in energy transfer.

## 1 Introduction

Light–matter interaction has always been a hot topic in fundamental research. Because of its potential applications in quantum information processing, optical sensing, and low threshold lasers, strong coupling between photons and excitons has attracted extensive attention [1], [2]. As the energy exchange rate between photons and excitons exceeds their average dissipation rate, strong exciton–photon coupling is achieved, forming the so-called exciton-polaritons with the feature of part light and part matter [3], [4]. Exciton-polarons are bosonic quasiparticles, which are of great importance in Bose–Einstein condensation [5], superfluids [6], and polariton lasing [7].

Transition metal dichalcogenide (TMDC) monolayer is considered as an ideal material for studying strong coupling due to large exciton binding energy (0.3−0.9 eV), large exciton transition dipole moment (56 D), and strong optical absorption (∼15 %) [8], [9]. So far, many studies have been devoted to the strong coupling between the excitons in a TMDC monolayer and the surface plasmon resonances excited in different nanocavities, such as single nanoparticles [10], [11], [12], [13], Fabry–Pérot cavities [14], nanoparticle-on-mirror systems [15], etc. In such plasmonic systems, a Rabi splitting energy as large as ∼240 meV has been demonstrated [16]. Apart from localized surface plasmons, strong exciton–plasmon coupling can also be realized between the propagating surface plasmon polaritons (SPPs) excited on a metal film and a TMDC monolayer. For example, the strong coupling between the SPPs excited on a thin Au film and exciton in a WS_2_ monolayer attached on the Au film leads to a Rabi splitting energy of ∼120 meV [17]. In addition, it was shown that the strong coupling between the transverse-electric (TE) polarized waves supported by a Si_3_N_4_/Ag heterostructure and the two excitons (A- and B-excitons) in a WS_2_ monolayer can create exciton–photon polaritons propagating on the WS_2_/Si_3_N_4_/Ag heterostructure [18].

Basically, the coupling between an optical cavity mode and multiple exciton resonances may lead to the hybridization of multiple excitons, achieving the energy exchange between the multiple excitons in the middle polariton branch. Such hybridization exhibits potential applications in energy capture, transfer, exchange, and storage [19]. Therefore, studying the hybridization of multiple excitons mediated by an optical mode is important to understand the energy transfer processes between semiconductors [20], [21]. In addition, the hybridization of multiple excitons may contribute to the development of new applications, such as an electrically injected polariton laser at room temperature. The concept of polariton condensation has been demonstrated by the cavity-mediated hybridization of GaAs and J-aggregate excitons [22]. It has been reported that the polariton-mediated coupling and energy transfer between excitonic states can be modulated by applying a magnetic field [23]. Moreover, energy transfer over mesoscopic (micron-length) distances by using the hybrid polariton states has the potential applications in solar cells and microfluidic devices [24]. So far, the strong coupling between the multiple excitons in a TMDC monolayer and plasmons has been demonstrated in different systems. For example, plasmon–exciton–trion polaritons have been revealed in a WS_2_ monolayer coupled to a plasmonic nanoantenna at low temperatures [25]. In addition, the A- and B-excitons in a few-layer MoS_2_ can be bridged by coupling with SPPs [26]. Recently, the strong coupling between the three excitons (neutral exciton, trion, and charged biexciton) in a WS_2_ monolayer and the plasmons in Ag nanocavities was demonstrated at low temperatures, forming plasmon–exciton–trion–charged biexciton hybrid states [27]. Apart from the hybridization of multiple excitons in the same material, it seems important to study the hybridization of multiple excitons in different materials, which may result in the energy exchange between different materials. Some initial studies have been carried out by using J-aggregates, quantum dots, and dye molecules [28], [29]. Recently, the strong coupling between the plasmons and excitons in J-aggregates and WS_2_ monolayer was observed, leading to a large double Rabi splitting (∼137 meV and ∼124 meV). In addition, the coherent energy exchange between the two excitons mediated by the plasmonic nanocavity was proved theoretically [30].

In recent years, a heterobilayer created by stacking two TMDC monolayers has attracted extensive attention due to its potential application in studying many rich physical phenomena in two-dimensional systems, such as interlayer excitons [31], Moiré patterns [32], [33], [34], [35], and valleytronics [36]. An enhancement factor of ∼15 was observed for the photoluminescence (PL) from the interlayer excitons in a WS_2_/MoS_2_ heterobilayer by using a nanocavity composed of an Ag cube and an Au film [37]. In addition, it was shown that the coupling between the excitons in a WS_2_/MoS_2_ heterobilayer and the Mie resonances supported by a Si nanoparticle can be employed to modify the emission of interlayer excitons [38]. However, most studies focus on the observation of interlayer excitons and enhanced PL from the heterobilayer and less attention has been paid to the coupling between plasmons and intralayer excitons in the heterobilayer.

Basically, the coupling strength between the plasmons supported by a nanocavity and the excitons in a two-dimensional material can be expressed as follows [39]:

(1) $$g\propto \mu \left|E\right|\sqrt{N},$$

where *μ* is the dipole moment of the excitons, *E* and *N* represent the vacuum electric field in the nanocavity and the number of excitons involved in the coupling. So far, many studies have been carried out on the achievement of strong plasmon–exciton coupling by enhancing the electric field in the nanocavity or by increasing the number of excitons involved in the coupling. On the other hand, the coupling strength can also be enhanced by increasing the dipole moment or oscillator strength of the excitons via laser excitation or gate voltage [10], [40].

In this article, we constructed a heterobilayer (MoS_2_/WS_2_ or WS_2_/MoS_2_) on a thin Au film or a Si_3_N_4_/Ag heterostructure and investigated numerically and experimentally the coupling between the surface wave (SPPs or TE waves) and the three excitons (A-exciton in WS_2_, A- and B-excitons in MoS_2_) in the heterobilayer. We observed anticrossing behaviors in the angle-resolved reflection (or the scattering) spectrum, and large Rabi splitting energies of ∼206.79 meV and ∼237.60 meV in the two coupling systems. We showed that the reduced damping rate and enhanced in-plane electric field of the TE wave lead to the strong coupling between the TE wave and the three excitons and the realization of exciton hybridization in the heterobilayer.

## 2 Results and discussion

**Figure 1:**
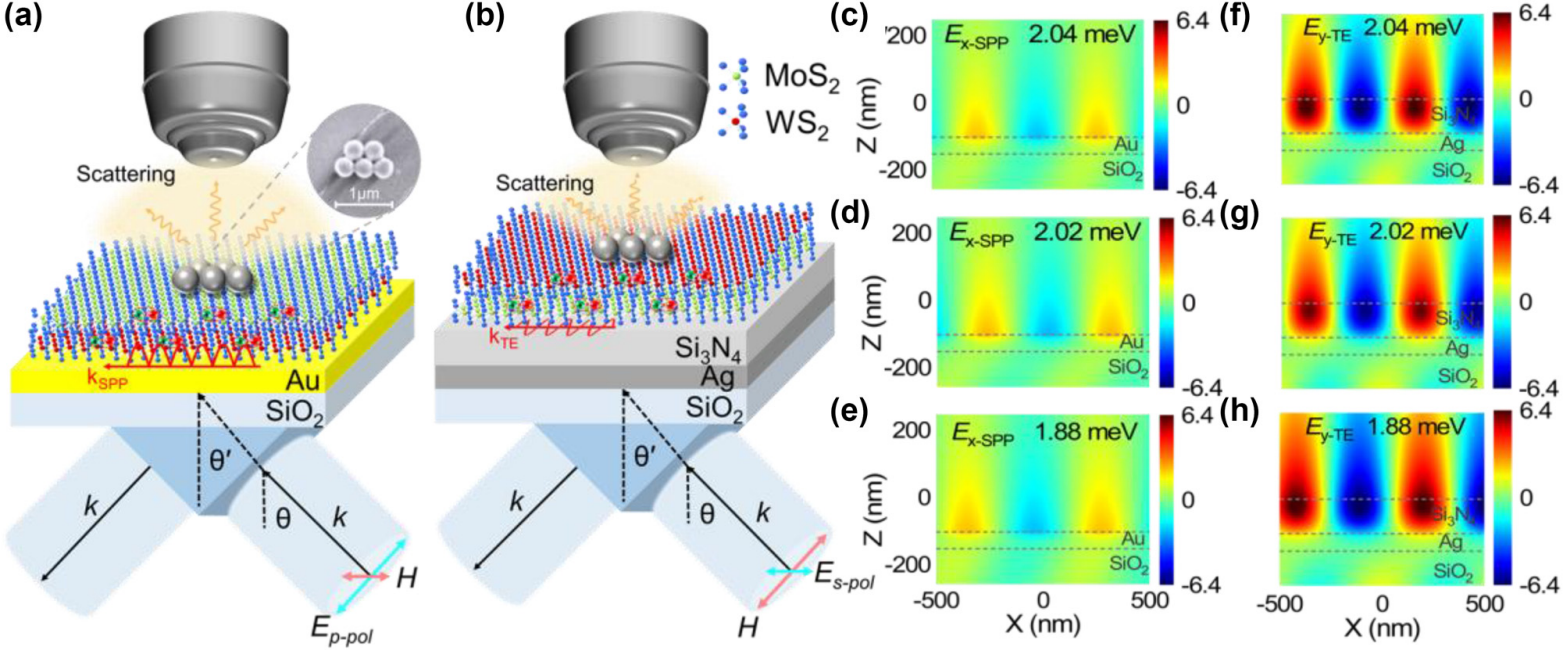
The coupling between the surface wave (SPP or TE wave) and three excitons in a heterobilayer (a) Schematic showing the coupling between the three excitons in a MoS_2_/WS_2_ heterobilayer and the SPP excited on the surface of an Au film by using *p*-polarized white light. (b) Schematic showing the coupling between the three excitons in WS_2_/MoS_2_ heterobilayer and the TE wave excited on the Si_3_N_4_/Ag heterostructure by using the *s*-polarized white light. In both cases, the surface waves (SPP or TE wave) are transferred into far-field radiations by using an oligomer of PS nanospheres and collected by the objective of a microscope. The SEM image of the oligomer of PS nanospheres is shown in the inset. (c–e) Electric field distributions (*E*_x_) of the SPP at the resonant energies of the three excitons in the heterobilayer. (f–h) Electric field distributions (*E*_y_) of the TE wave at the resonant energies of the three excitons in the heterobilayer.

In Figure 1a, we show schematically the coupling between the SPPs excited on a thin Au film (or an Au/SiO_2_ substrate) and the three excitons in a MoS_2_/WS_2_ heterobilayer attached on the Au/SiO_2_ substrate. The SPPs are generated by using *p*-polarized light in the Kretschmann–Raether (K–R) configuration. Similarly, one can excite TE waves on a Si_3_N_4_/Ag heterostructure by using *s*-polarized light and investigate the coupling between the TE waves and the three excitons in a WS_2_/MoS_2_ heterobilayer, as schematically shown in Figure 1b. In both cases, the coupling between the surface waves (SPPs or TE waves) and the excitons are reflected in the angle-resolved reflection or scattering spectra. Basically, the electromagnetic field distribution of a TE wave is different from that of a SPP. It has been revealed that the electric field and magnetic fields of the TE wave are localized on the upper surface of the Si_3_N_4_ layer and the interface between the Si_3_N_4_ layer and the Ag film, respectively [41]. In comparison, electric and magnetic fields of the SPP, which is a transverse magnetic (TM) wave, are localized on the surface of the metal film. It has been known that the separation of electric and magnetic fields in a photonic crystal will lead to the strong localization of electromagnetic wave (or a small group velocity) [42], [43]. Therefore, the spatial separation of electric and magnetic fields in the TE wave results in a small radiation loss (or a narrow linewidth) of the TE wave. In addition, the in-plane electric field (*E*_y_) of the TE wave is enhanced as compared with the corresponding TM wave (*E*_x_). These features of the TE wave are beneficial for realizing strong coupling between the TE wave and the three excitons in the heterobilayer. Since the resonant energies of the SPP and TE wave are closely related to the angle of the incident light, one can easily tune the resonant energies of the two surface waves in the range of 1.75–2.25 eV by simply adjusting the incident angle. This feature offers us an opportunity for examining the coupling between the surface waves and various excitons in the heterobilayer. Therefore, we numerically and experimentally compared the coupling of TE and TM waves (SPP) with the three excitons in a heterobilayer, respectively. Since the size of the WS_2_/MoS_2_ heterobilayer (∼20 μm) is much smaller than that of incident light beam, it remains a big challenge to measure the reflection spectrum of the heterobilayer on the Au/SiO_2_ substrate or the Si_3_N_4_/Ag heterostructure. In experiments, we intentionally introduce oligomers of PS nanospheres on the heterobilayer as the scatters to transfer the surface wave (SPP or TE wave) into a far-field radiation. The information of the coupling between the surface wave and the excitons in the heterobilayer can be extracted from the scattering spectra of oligomers of PS nanospheres, as schematically shown in Figure 1a and b. Based on numerical calculation, it was found that the ordering of MoS_2_ and WS_2_ monolayer in the heterobilayer has negligible influence on the coupling between the surface wave and the excitons (see Supplementary Note 1). Therefore, we investigated the coupling between the surface wave and the three excitons in the heterobilayer by measuring the scattering spectra of the existing samples (PS/MoS_2_/WS_2_/Au and PS/WS_2_/MoS_2_/Si_3_N_4_/Ag).

In this work, the excitons in the heterobilayer involved in the coupling with surface waves include A-exciton (X_A1_) in WS_2_ monolayer (∼2.016 eV), A- (X_A2_) (∼1.879 eV) and B-excitons (X_B_) (∼2.043 eV) in MoS_2_ monolayer. The oscillator strength of the exciton is proportional to the integrated area of the imaginary part of the dielectric function [44]. It is reported that the imaginary part of X_A1_ in WS_2_ monolayer is stronger than that of X_A2_ in MoS_2_ monolayer [44]. Therefore, the oscillator strength of X_A1_ is larger than that of X_A2_. Since the coupling strength *g* is proportional to the electric field in the optical cavity and the excitons in TMDC monolayer are mainly oriented in the XY plane, we examined only the in-plane electric field components of the two surface waves, which can interact effectively with the excitons. Therefore, we calculated the x component of the electric field (*E*_x_) for the SPP and the y component of the electric field (*E*_y_) for the TE wave. Since the SPP and TE wave are generated by *p*- and *s*-polarized light, respectively, we calculated the x component of the electric field (*E*_x_) for the SPP and the y component of the electric field (*E*_y_) for the TE wave. In Figure 1c–e, we present the electric field distribution (*E*_x_) of the SPP at the resonant energies of the three excitons. It can be seen that the electric field of the SPP is located on the upper surface of the Au film, which facilitates the coupling between the SPP and the excitons in the heterobilayer. The amplitude of electric field for the SPP at the three exciton resonances is estimated to be ∼2.4 (see also Supplementary Note 2). In Figure 1f–h, we present the electric field distributions (*E*_y_) calculated for the TE wave at three exciton resonances. The enhancement factors for the electric field on the surface of the Si_3_N_4_ layer at the three exciton resonances are found to be ∼6.4 (see also Supplementary Note 3). These values are larger than those observed for the SPP, implying that the coupling strength between TE wave and three excitons is greater than that of the SPP. In addition, the coupling strength is inversely proportional to the square root of the mode volume (*V*) (i.e., $g\propto 1/\sqrt{V}$ ). Since the mode volume of the TE wave is smaller than that of the SPP (see Supplementary Note 4), it implies that a stronger photon–exciton coupling can be achieved between the TE wave and the three excitons in the heterobilayer.

**Figure 2:**
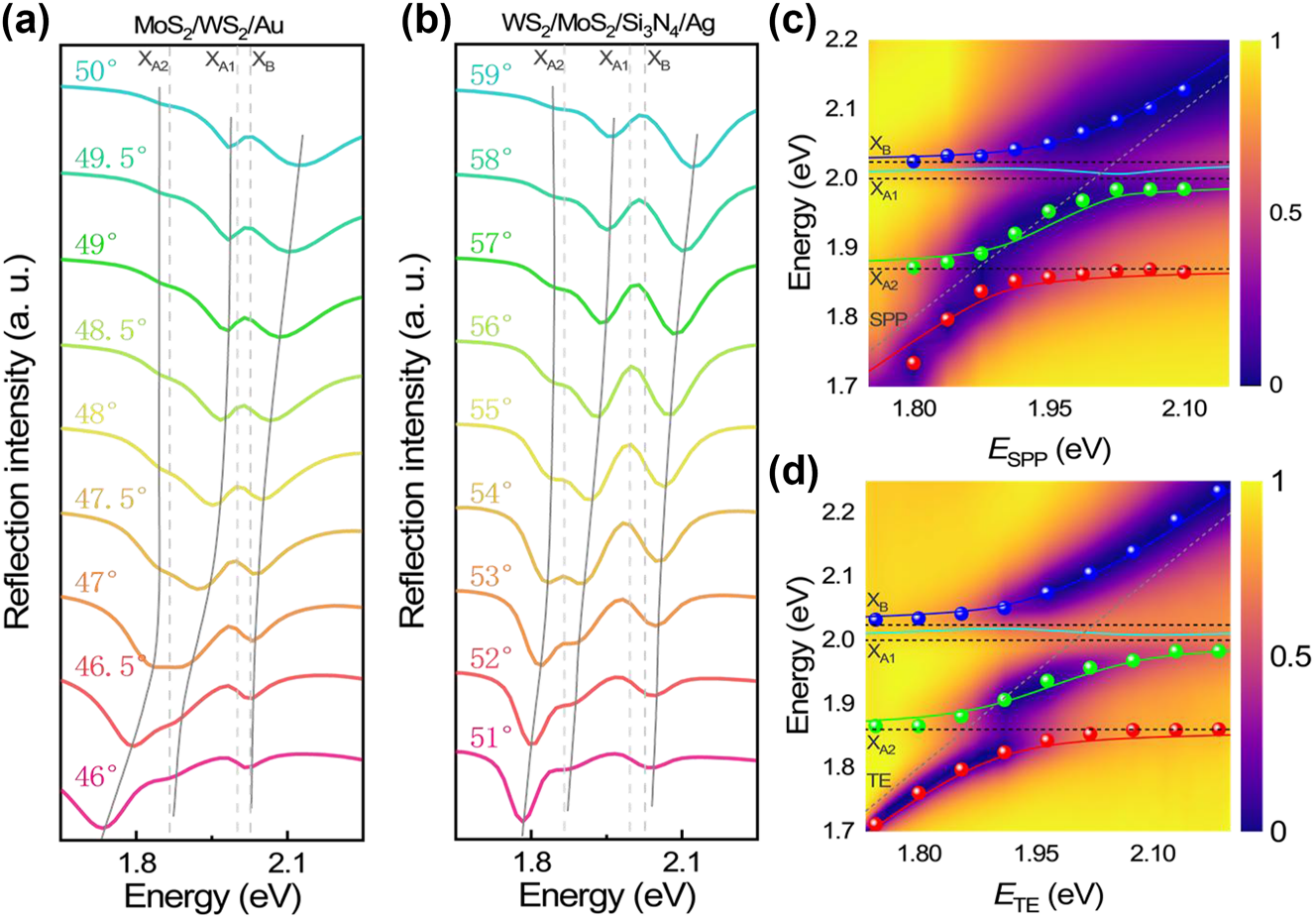
Reflection spectra calculated for a heterobilayer (MoS_2_/WS_2_ or WS_2_/MoS_2_) attached on an Au film (a) and a Si_3_N_4_/Ag heterostructure (b) at different incident angles. The resonant energies of the three excitons are indicated by the dashed lines. (c, d) Dispersion relations (solid symbols) of the hybrid states extracted from the reflection spectra shown in (a) and (b). Also shown are the fitting results of the dispersion relations based on the Hamiltonian (solid lines). The resonant energies of the surface waves and the three excitons are indicated by dashed lines.

In Figure S5, one can see that the linewidth of the TE wave extracted from the reflection spectrum is much narrower as compared with that of the SPP. This feature indicates that the strong coupling criterion is easier to be fulfilled for the coupling between the TE wave and the three excitons. As shown in Figure 2a, we show the reflection spectra calculated for MoS_2_/WS_2_/Au excited by using *p*-polarized light with different incident angles. One can see three dips (marked by solid lines) and two peaks (marked by dashed lines) in the reflection spectra. Since the energy of X_A1_ is very close to that of X_B_, it is difficult to distinguish the Rabi splitting resulting from the coupling of these two excitons in the reflection spectrum. Here, the two reflection peaks correspond to the three exciton resonances while the two valleys originate from hybrid states generated by the coupling between the SPP and the excitons. It can be seen that the resonant energy of the SPP is blueshifted with increasing incident angle (see Supplementary Note 6) and the reflection spectra exhibit anticrossing behaviors at the resonant energies of X_A1_ and X_A2_. It is noticed that the Rabi splitting resulting from the coupling between the SPP and X_A1_ exciton is more pronounced than that between the SPP and X_A2_ exciton. It is because that the oscillator strength of X_A1_ is larger than that of X_A2_ [27], [45]. Figure 2b shows the reflection spectra calculated for the WS_2_/MoS_2_/Si_3_N_4_/Ag structure excited by using *s*-polarized light with different incident angles. Similar to the SPP, the resonant energy of the TE wave is blueshifted with increasing incident angle (see Supplementary Note 7). In addition, Rabi splitting is observed at the three exciton resonances. As compared with the SPP, the Rabi splitting originating from the coupling between the TE wave and the exciton is more pronounced due to the larger enhancement in the electric field (see Figure 1f–h).

Based on the coupled oscillator model, the coupling between the surface wave (SPP or TE wave) and the three excitons can be described by using a 4 × 4 Hamiltonian expressed as follows [27]:

(2) $$H=\left(\begin{array}{cccc} E_{\mathrm{cav}}-i\frac{\gamma_{\mathrm{cav}}}{2} & g_{\mathrm{cav-XA1}} & g_{\mathrm{cav-XA2}} & g_{\mathrm{cav-XB}}\\ g_{\mathrm{cav-XA1}} & E_{\mathrm{XA1}}-i\frac{\gamma_{\mathrm{XA1}}}{2} & 0 & 0\\ g_{\mathrm{cav-XA2}} & 0 & E_{\mathrm{XA2}}-i\frac{\gamma_{\mathrm{XA2}}}{2} & 0\\ g_{\mathrm{cav-XB}} & 0 & 0 & E_{XB}-i\frac{\gamma_{XB}}{2} \end{array}\right),$$

where *E*_cav_ (*cav* = SPP, TE), *E*_X*i*_ (*i* = A1, A2, B) represent the uncoupled resonant energies of the surface wave and three excitons, *γ*_cav_, *γ*_X*i*_ represent the dissipation rates of the surface wave and the three excitons, and *g*_cav-X*i*_ represent the coupling strengths between the surface wave and the three excitons, respectively. The resonant energies and linewidths of the surface waves and two excitons can be obtained from numerical simulations and previous literatures [16], [18]. The diagonalization of Hamiltonian yields the eigenenergies of the four hybrid states. The corresponding Hopfield coefficients indicate the contributions of the surface wave and the three excitons. Basically, the resonant energies of the hybrid states, which are referred to as the upper, two middle, and lower polaritons branches (UPB, MPB1, MBP2, and LPB), can be extracted from the reflection dips (or scattering peaks) observed in the reflection (or scattering) spectrum of the coupled system. In Figure 2c, we present the two-dimensional reflection spectra calculated for the MoS_2_/WS_2_/Au, which is excited by using *p*-polarized light with different incident angles (corresponding to the SPPs with different energies). In Figure 2c, the dispersion relations extracted from the reflection spectra are represented by solid symbols while the fitting results based on the Hamiltonian are represented by solid curves. One can clearly identify two anticrossing behaviors at the energies of X_A1_ and X_A2_, which give the dispersion relations of the hybrid states formed by the coupling between the SPP and the three exciton resonances. Although MPB1 can be derived from the Hamiltonian (cyan curve), it cannot be extracted from the reflection and scattering spectra (cyan symbols) because the energy of X_A1_ (∼2.02 eV) is very close to that of X_B_ (∼2.04 eV). In this case, the Rabi splitting energy is derived to be ∼198.05 meV. In Figure 2d, we present the two-dimensional reflection spectra calculated for the WS_2_/MoS_2_/Si_3_N_4_/Ag structure, which is excited by using *s*-polarized light with different incident angles (corresponding to the TEs with different energies). Similarly, the couplings between the TE waves and the three exciton resonances are manifested as two anticrossing behaviors in the angle-resolved reflection spectra. It can be seen that the dispersion relations extracted from the reflection spectra (solid symbols) are well fitted by the calculation results based on the Hamiltonian (solid curves). In this case, a larger Rabi splitting energy of ∼234.85 meV is observed for the TE wave.

**Figure 3:**
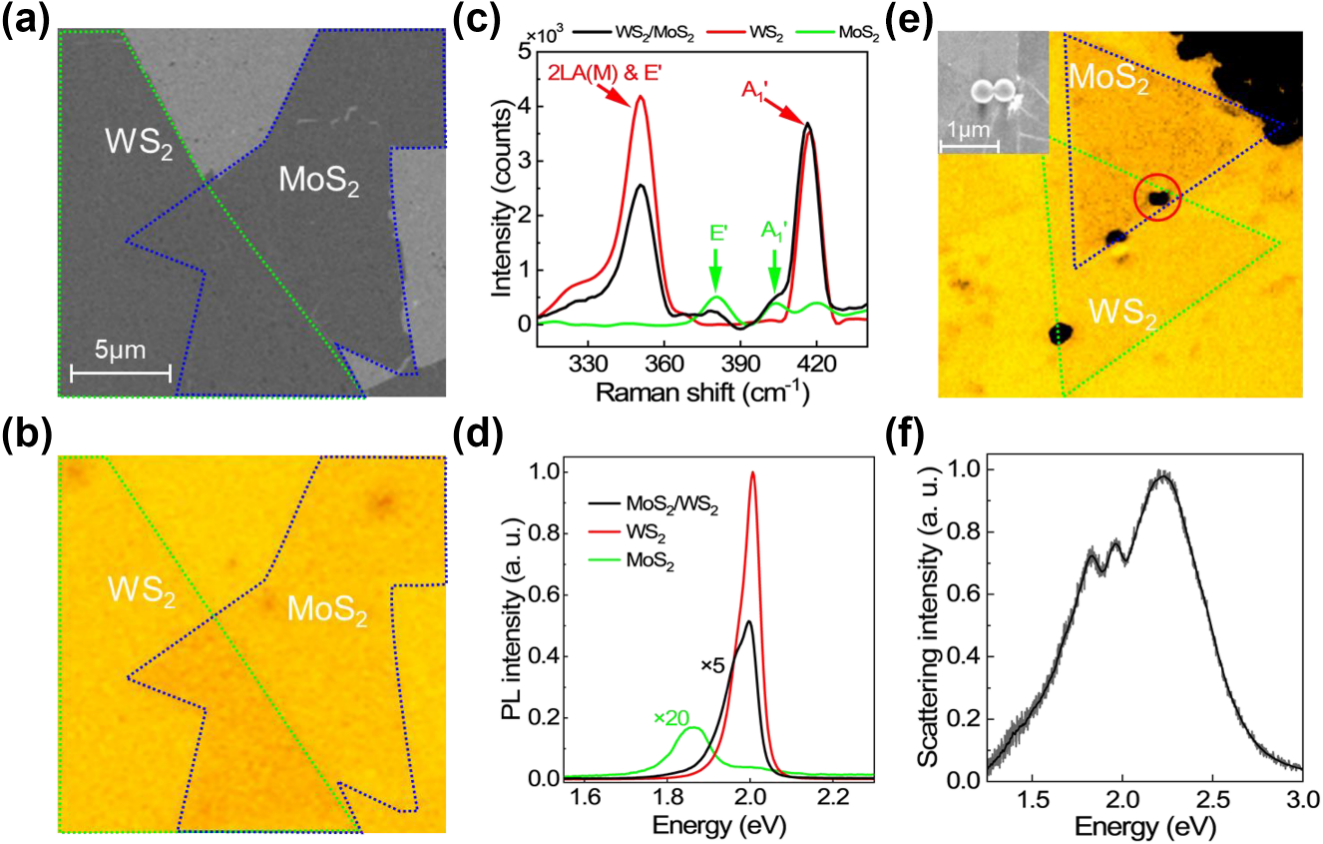
SEM (a) and bright-field microscope (b) images of a typical MoS_2_/WS_2_ heterobilayer. (c) Raman scattering spectra measured for the WS_2_/MoS_2_ heterobilayer (black curve), the WS_2_ monolayer (red curve), and MoS_2_ monolayer (green curve). (d) PL spectra measured for the MoS_2_/WS_2_ heterobilayer (black curve), the WS_2_ monolayer (red curve), and MoS_2_ monolayer (green curve). (e) Bright-field microscope image of the dimer of PS nanospheres located on the WS_2_/MoS_2_ heterobilayer. The corresponding SEM image of the dimer of PS nanospheres is shown in the inset. (f) Forward scattering spectrum measured for the oligomer of PS nanospheres located on the WS_2_/MoS_2_/Si_3_N_4_/Ag structure.

In order to validate the simulation results, we investigated experimentally the two coupling systems by measuring the scattering spectra of PS oligomers. As discussed above, we introduced oligomers of PS nanospheres as scatters to transfer the surface wave (SPP or TE wave) into a far-field radiation. In Figure 3a and b, we present the scanning electron microscope (SEM) and bright-field microscope images of a MoS_2_/WS_2_ heterobilayer. The WS_2_ and MoS_2_ monolayers were grown by chemical vapor deposition (CVD) and subsequently transferred onto a substrate (Au/SiO_2_ or Si_3_N_4_/Ag/SiO_2_) by using a wet-transfer approach (see the Materials for the details). Figure 3c shows the Raman spectra measured for the WS_2_ monolayer, MoS_2_ monolayer, and WS_2_/MoS_2_ heterobilayer. The four peaks observed at 350, 380, 405, and 415 cm^−1^ are attributed to the out-of-plane E′ and in-plane A_1_′ modes of WS_2_ and MoS_2_ monolayer. It indicates that the heterobilayer is composed of WS_2_ and MoS_2_ monolayers [46]. In Figure 3d, we show the PL spectra measured for the WS_2_ monolayer, MoS_2_ monolayer, and WS_2_/MoS_2_ heterobilayer. Due to interlayer charge transfer, the PL intensity of the MoS_2_/WS_2_ heterobilayer is dramatically reduced as compared with that of the WS_2_ monolayer [47]. Since the PL intensity of MoS_2_ monolayer is lower by an order of magnitude than that of WS_2_ monolayer, the PL peak of MoS_2_ monolayer is not observed [48]. In Figure 3e, we show the bright-field microscope image of an oligomer of PS nanospheres located on the WS_2_/MoS_2_ heterobilayer. Based on the SEM image (see the inset), it is a dimer of PS nanospheres. In Figure 3f, we present the forward scattering spectrum measured for the oligomer of PS nanospheres. It appears as a broadband without any resonances in the visible light spectrum. Thus, it has no influence on the coupling between the surface wave and the excitons in the heterobilayer. The two dips observed in scattering spectrum correspond to X_A1_ and X_A2_ in WS_2_ and MoS_2_ monolayer (also see Supplementary Note 8).

**Figure 4:**
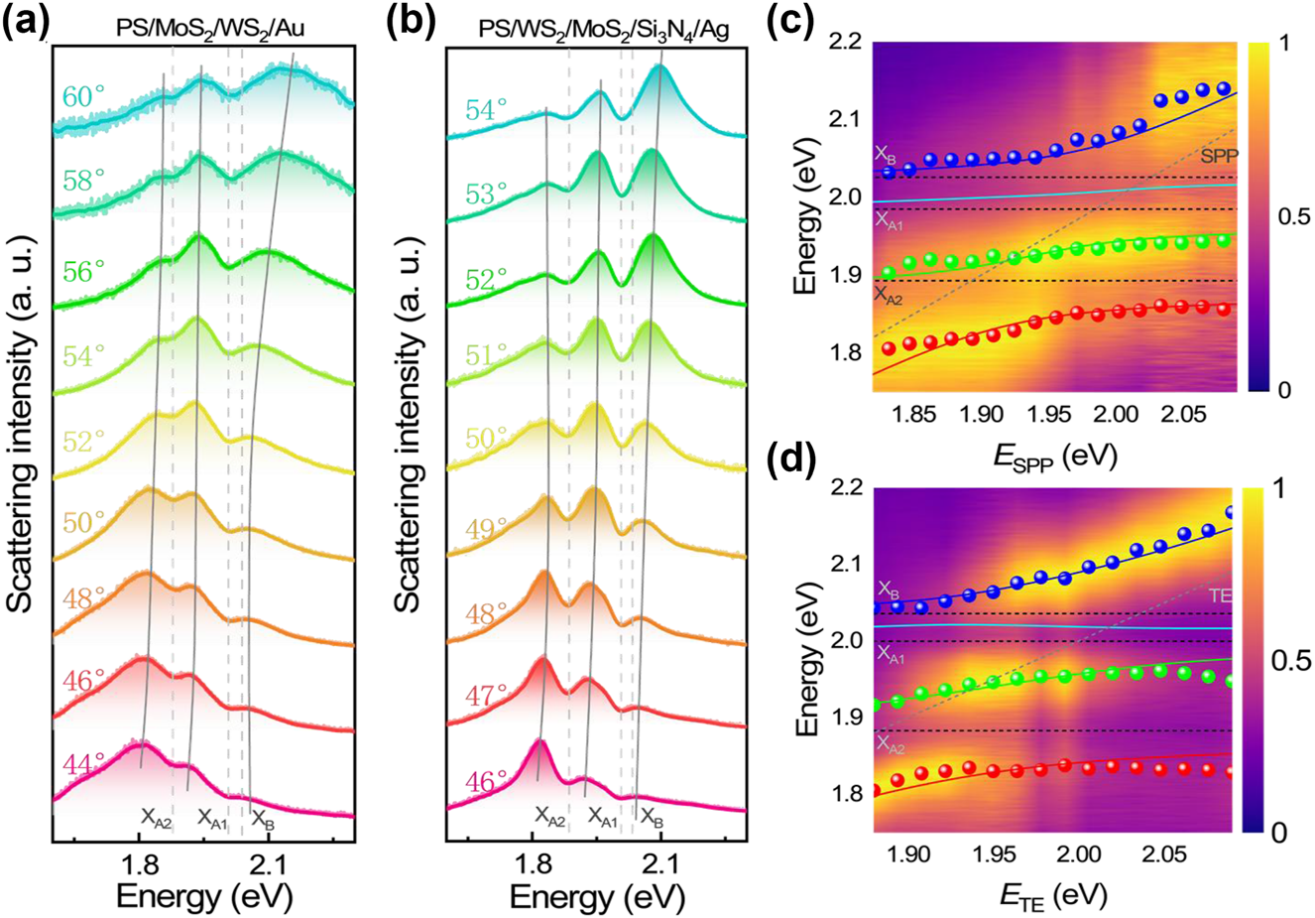
Scattering spectra measured for an oligomer of PS nanospheres placed on a heterobilayer attached on an Au film (a) and a Si_3_N_4_/Ag heterostructure (b) at different incident angles. The resonant energies of the three excitons are indicated by the dashed lines. (c, d) Dispersion relations (solid symbols) of the hybrid states extracted from the scattering spectra shown in (a) and (b). Also shown are the fitting results of the dispersion relations based on the Hamiltonian (solid lines). The resonant energies of the surface waves and the three excitons are indicated by dashed lines.

In Figure 4a, we present the scattering spectra of an oligomer of PS nanospheres located on a MoS_2_/WS_2_/Au structure, which is excited by using *p*-polarized light with different incident angles (see also Supplementary Note 9, 10). In all cases, one can identify three scattering peaks (marked by solid lines) in the scattering spectra, corresponding to the hybrid states formed by the coupling between the SPP and the three exciton resonances. The three exciton resonances appear as scattering dips (marked by dashed lines) in the scattering spectrum. At small incident angles, the two dips are relatively shallow and LPB is dominant because the SPP is located on the low-energy side of the exciton resonance. With increasing incident angle, the SPP is gradually shifted to high energies and the scattering spectrum becomes eventually dominated by UPB. In Figure 4c, we plot the two-dimensional scattering spectra measured for the oligomer of PS nanospheres located on the MoS_2_/WS_2_/Au structure at different incident angles, corresponding to the SPPs with different energies (*E*_SPP_). The dispersion relations of the hybrid states extracted from the scattering spectra (see Figure 4a) are represented by solid symbols while the fitting results based on Hamiltonian are represented by solid curves. It can be seen that the dispersion relations extracted from the scattering spectra (solid symbols) are well fitted by the calculation results (solid curves). In this case, a Rabi splitting energy (Ω_SPP_) of ∼206.79 meV is observed. In order to find out whether the coupling between the SPP and the three excitons enters the strong coupling regime, we derived the three coupling strengths *g*_SPP-X*i*_ = 69, 52, 35 meV from the fitting results. The dissipation rates of the SPP (*γ*_SPP_) and the three exciton resonances (*γ*_*i*_) are found to be ∼220, ∼33, ∼98, ∼132 meV, respectively. It was found that the strong coupling criterion (*g*_SPP-X*i*_ > (*γ*_SPP_ + *γ*_X*i*_)/4) is not satisfied for X_A2_ and X_B_ due to the large dissipation rates of the SPP and two excitons in MoS_2_ monolayer [27]. In this case, only the coupling between the SPP and X_A1_ enters the strong coupling regime owing to the large oscillator strength and small dissipation rate of X_A1_ in WS_2_ monolayer. In comparison, the coupling between SPP and two excitons in MoS_2_ monolayer only reaches intermediate coupling (i.e., *g*_SPP-X*i*_ > (*γ*_SPP_ − *γ*_X*i*_)/4). In order to enhance the coupling strength between the SPP and the three excitons, we tried to increase the number of excitons involved in the coupling by using a MoS_2_/WS_2_ heterostructure composed of few-layer MoS_2_ and WS_2_. In this way, the Rabi splitting energy (Ω_SPP_) is increased to ∼261.20 meV and the coupling strengths are further enhanced to *g*_SPP-X*i*_ = 88, 57, 50 meV (see Supplementary Note 11). Unfortunately, the strong coupling is still not achieved by using the MoS_2_/WS_2_ heterostructure composed of few-layer MoS_2_ and WS_2_.

In Figure 4b, we present the scattering spectra of an oligomer of PS nanospheres located on a WS_2_/MoS_2_/Si_3_N_4_/Ag structure, which is excited by using *s*-polarized light with different incident angles (see also Supplementary Note 9, 12). Similarly, one can observe three scattering peaks and two scattering dips in all scattering spectra, which originate from the coupling between the TE wave and the three excitons. As compared with the coupling between the SPP and the three excitons, the scattering peaks representing the hybrid states and the scattering dips corresponding to the exciton resonances become more pronounced. In Figure 4d, we plot the two-dimensional scattering spectra measured for the oligomer of PS nanospheres located on the WS_2_/MoS_2_/Si_3_N_4_/Ag structure at different incident angles, corresponding to the TE waves with different energies (*E*_TE_). The dispersion relations of the hybrid states extracted from the scattering spectra (see Figure 4b) are represented by solid symbols while the fitting results are also provided by solid curves. In this case, the Rabi splitting energy is found to be Ω_TE_ ∼237.60 meV and the coupling strengths are derived to be *g*_TE-X*i*_ = 57, 66, 50 meV. The Rabi splitting energy observed for the TE wave is larger than that for the SPP. On the other hand, the dissipation rate of the TE wave (∼64 meV) is much smaller than that of the SPP (∼220 meV). Therefore, the coupling strengths between TE wave and the three exciton resonances satisfy the criterion of strong coupling (i.e., *g*_TE-X*i*_ > (*γ*_TE_ + *γ*_X*i*_)/4).

**Figure 5:**
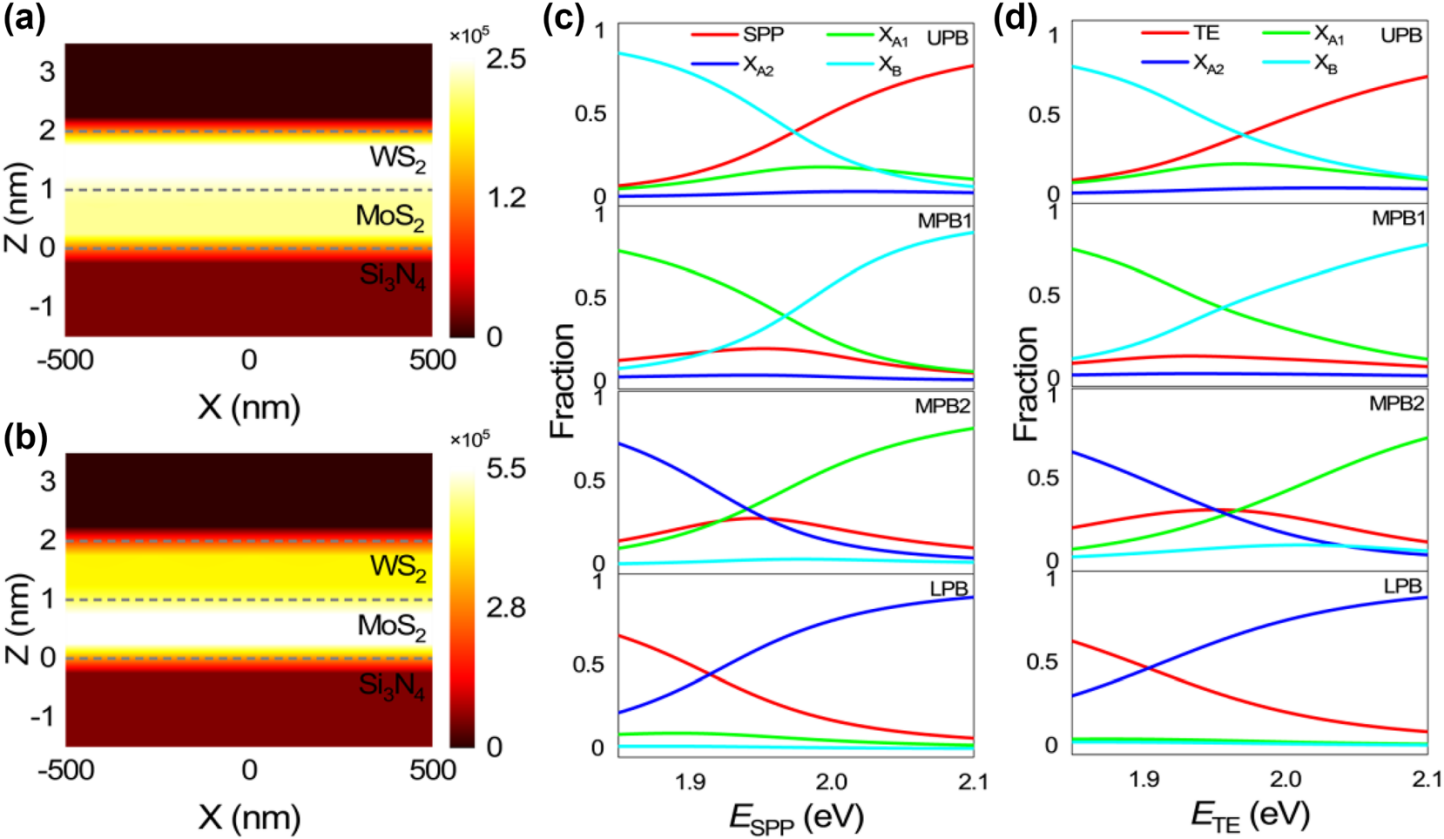
The hybridization of three excitons in a heterobilayer mediated by the surface waves (a) Current density distribution in a WS_2_/MoS_2_ heterobilayer on the Si_3_N_4_/Ag heterostructure calculated at the energy of X_A1_ (2.02 eV) and the incident angle of 56°. (b) Current density distribution in a WS_2_/MoS_2_ heterobilayer on the Si_3_N_4_/Ag heterostructure calculated at the energy of X_A2_ (1.88 eV) and the incident angle of 54°. (c, d) Hopfield coefficients derived for the surface wave (SPP or TE wave) and three excitons in UPB, MPB1, MPB2, and LPB as a function of the energy of the surface wave (SPP or TE wave).

When strong coupling occurs, the hybridization of multiple excitons mediated by the optical mode may result in the efficient energy transfer between them [49]. Apparently, the hybridization of multiple excitons is reflected in the contributions of the excitons in MPB. In Figure S13, we show the current density distribution in a Si_3_N_4_/Ag structure calculated for the TE wave at two incident angles. One can see that the current densities are mainly distributed in the Si_3_N_4_ layer in both cases. In Figure 5a, we present the current density distribution calculated for a WS_2_/MoS_2_/Si_3_N_4_/Ag structure at an incident angle of 56°, corresponding to the energy of X_A1_. Interestingly, it is found that the current density becomes concentrated in the WS_2_ monolayer, implying the energy transfer from the Si_3_N_4_ layer to the WS_2_ monolayer [50]. Similarly, the current density is concentrated in the MoS_2_ monolayer when the TE wave is resonant with X_A2_ at an incident angle of 54°, as shown in Figure 5b. Additionally, the energy transfer is also reflected in the reduced electric field distribution in the Si_3_N_4_/Ag structure after the introduction of the WS_2_/MoS_2_ heterobilayer (see Supplementary Note 3). Therefore, one can manipulate the energy transfer from the Si_3_N_4_ layer to the WS_2_ or MoS_2_ monolayer by simply adjusting the incident angle. Based on eq. (2), we also calculated the contributions of the surface wave (SPP or TE wave) and three excitons in each hybrid state, which are reflected in the Hopfield coefficients as a function of the energy of the surface wave, as shown in Figure 5c and d. In both cases, LPBs are mainly composed of the surface wave and X_A2_. As the energy of the surface wave increases, the fraction of the surface wave decreases while that of X_A2_ increases. In comparison, UPBs are mainly composed of the surface wave and two excitons (X_A1_ and X_B_). With increasing the energy of the surface wave, the fraction of the surface wave increases while that of X_B_ decreases. When the fractions of the surface wave and X_B_ become equal, the fraction of X_A1_ reaches its maximum, implying the hybridization of X_A1_ and X_B_. In MPB1, the fraction of X_A2_ is quite small (∼3.21 % in Figure 5c and ∼2.67 % in Figure 5d) because its energy is far away from MPB1. In contrast, it can be seen that MBP1 is dominated by X_A1_ and X_B_ with similar contributions while the contribution of the surface wave is small. In addition, the optical absorption of the excitons usually leads to dips in the scattering spectrum (see Figure 3f). This is the reason why MPB1 cannot be revealed in the reflection or scattering spectrum and only a Rabi splitting energy is observed. In MBP2, X_A1_ and X_A2_ have the equal contributions (∼34.07 %) while the fractions of the SPP and X_B_ are ∼28.38 % and ∼3.48 % at *E*_SPP_ = 1.94 eV, respectively. In comparison, TE wave, X_A1_, and X_A2_ have the similar contributions (∼31.89 %, ∼29.48 %, ∼29.48 %) while the fraction of X_B_ is ∼9.28 % at *E*_TE_ = 1.96 eV. It implies that the TE wave can effectively mediate the hybridization of the three excitons, which suggests the possibility of the energy transfer between the three excitons in MPB.

## 3 Conclusions

In summary, we have systematically investigated the coupling between the surface wave (SPP or TE wave) and three excitons (X_A1_, X_A2_, X_B_) in a MoS_2_/WS_2_ (or WS_2_/MoS_2_) heterobilayer by using two coupling systems (MoS_2_/WS_2_/Au and WS_2_/MoS_2_/Si_3_N_4_/Ag structures). We found that the coupling between the surface wave and three excitons is revealed as anticrossing behaviors in the angle-resolved reflection and scattering spectra. We observed a Rabi splitting energy of Ω_SPP_ ∼206.79 meV for the SPP and an enhanced Rabi splitting energy of Ω_TE_ ∼237.60 meV for the TE wave due to its reduced dissipation rate and enhanced electric field. The similar contributions of the TE wave, X_A1_, and X_A2_ in MPB2 indicate clearly the hybridization of the three excitons mediated by the TE wave. The exciton hybridization resulting from the strong coupling between the TE wave and three excitons in a heterobilayer demonstrated in this work suggests the potential applications of energy transfer between the multiple excitons in TMDC heterobilayer in the development of novel photonic devices.

## 4 Methods

### 4.1 Sample preparation

In this work, the thicknesses of the Au film, Ag film, and Si_3_N_4_ film were designed to be ∼50 nm, ∼50 nm, and ∼100 nm, respectively. The WS_2_ and MoS_2_ monolayers used in this work were purchased from SixCarbon Technology Shenzhen. The WS_2_ and MoS_2_ monolayers grown by CVD on a SiO_2_/Si substrate were transferred onto an Au/SiO_2_ and a Si_3_N_4_/Ag/SiO_2_ substrate by using the following procedure. First, a thin film of poly (methyl methacrylate) (PMMA) was spin-coated onto a WS_2_ monolayer. Then, the PMMA/WS_2_ film was separated from the SiO_2_/Si substrate by etching with KOH (2 mol L^−1^) at 80 °C. After that, the residual KOH was removed by deionized water, and the PMMA/WS_2_ film was transferred onto an Au/SiO_2_. Finally, the PMMA was dissolved by acetone. By using the same method, a MoS_2_ monolayer was transferred onto the WS_2_ monolayer, creating a MoS_2_/WS_2_ on the Au/SiO_2_ substrate. Similarly, a WS_2_/MoS_2_ heterobilayer was formed on the Si_3_N_4_/Ag/SiO_2_ substrate. The aqueous solution of polystyrene (PS) nanospheres was dropped and dried onto the heterobilayer, forming oligomers of PS nanospheres.

### 4.2 Optical characterization

In this work, SPP and TE wave can be generated on the surface of an Au film or a Si_3_N_4_/Ag heterostructure by using the K–R configuration, as shown in Figure 1a and b. A prism made of SiO_2_ (K9 glass) was used to couple the *p*- and *s*-polarized white light into the optical system through total internal reflection. An inverted microscope (Axio Observer A1, Zeiss), a spectrometer (SR-500i-B1, Andor), and a color charge-coupled device (CCD) (DS-Ri2, Nikon) were used to obtain the scattering spectra and dark-field images of the PS nanospheres.

### 4.3 Numerical simulations

In this work, the numerical simulations were performed by using the finite-difference time-domain (FDTD) technique. The dielectric constants of Ag and Au were taken from Johnson and Christy [51]. The dielectric constants of MoS_2_ and WS_2_ monolayers were obtained from literature [44]. The refractive index of the surrounding media was chosen to be 1.0. In the calculation, the thickness of the MoS_2_ and WS_2_ monolayers was chosen to be 1.0 nm. A mesh size as small as 0.5 nm was used to ensure the convergence of numerical simulations and the achievement of accurate results.

## Supplementary Material

Supplementary Material Details
