# Supplementary material for: Exciton hybridization in a WS2/MoS2 heterobilayer mediated by a surface wave via strong photon–exciton coupling
Source: Nanophotonics. 2025 Mar 14;14(5):601–11. doi: 10.1515/nanoph-2024-0737 (PMC11953721; doi:10.1515/nanoph-2024-0737)
Supplement: Supplementary file 1 — Supplementary Material Details [file j_nanoph-2024-0737_suppl_001.docx]

Supporting Information

**Exciton hybridization in a** **WS_2_/MoS_2_ heterobilayer mediated by a surface wave via strong photon-exciton coupling**

Junxuan Yi,^1^ Shimei Liu,^1,*^ Shulei Li,^2^ Weichen He,^3,4^ Zuxin Chen^5^ and Sheng Lan^1,*^

^1^ Guangdong Provincial Key Laboratory of Nanophotonic Functional Materials and Devices, School of Optoelectronic Science and Engineering, South China Normal University, Guangzhou 510006, China

^2^ School of Optoelectronic Engineering, Guangdong Polytechnic Normal University, Guangzhou 510665, China

^3^ GBA Branch of Aerospace Information Research Institute, Chinese Academy of Sciences, Guangzhou, 510700, China

^4^ Guangdong Provincial Key Laboratory of Terahertz Quantum Electromagnetics, Guangzhou, 510700, China

^5^ School of Electronic Science and Engineering (School of Microelectronics), South China Normal University, Foshan, 528225, China

* Corresponding author: [mn551173@foxmail.com](mailto:mn551173@foxmail.com) (S. Liu), [slan@scnu.edu.cn](mailto:slan@scnu.edu.cn) (S. Lan)

Supplementary Note 1

In Figure S1, we present the reflection spectra calculated for the MoS_2_/WS_2_/Au and WS_2_/MoS_2_/Au excited by using *p*-polarized light, as well as the MoS_2_/WS_2_/Si_3_N_4_/Ag and WS_2_/MoS_2_/Si_3_N_4_/Ag structure excited by using *s*-polarized light, respectively. One can see that the reflection spectrum of the MoS_2_/WS_2_/Au is the same as that of the WS_2_/MoS_2_/Au. Moreover, there is not significant difference between the reflection spectrum of the WS_2_/MoS_2_/Si_3_N_4_/Ag and that of the MoS_2_/WS_2_/Si_3_N_4_/Ag. It indicates that the ordering of MoS_2_ and WS_2_ monolayer in the heterobilayer has negligible influence on the coupling between the surface wave and the excitons. For the convenience of the experiments, we investigated the cuopling between the surface wave and the three excitons in the heterobilayer by measuring the scattering spectra of the existing samples (PS/MoS_2_/WS_2_/Au and PS/WS_2_/MoS_2_/Si_3_N_4_/Ag).


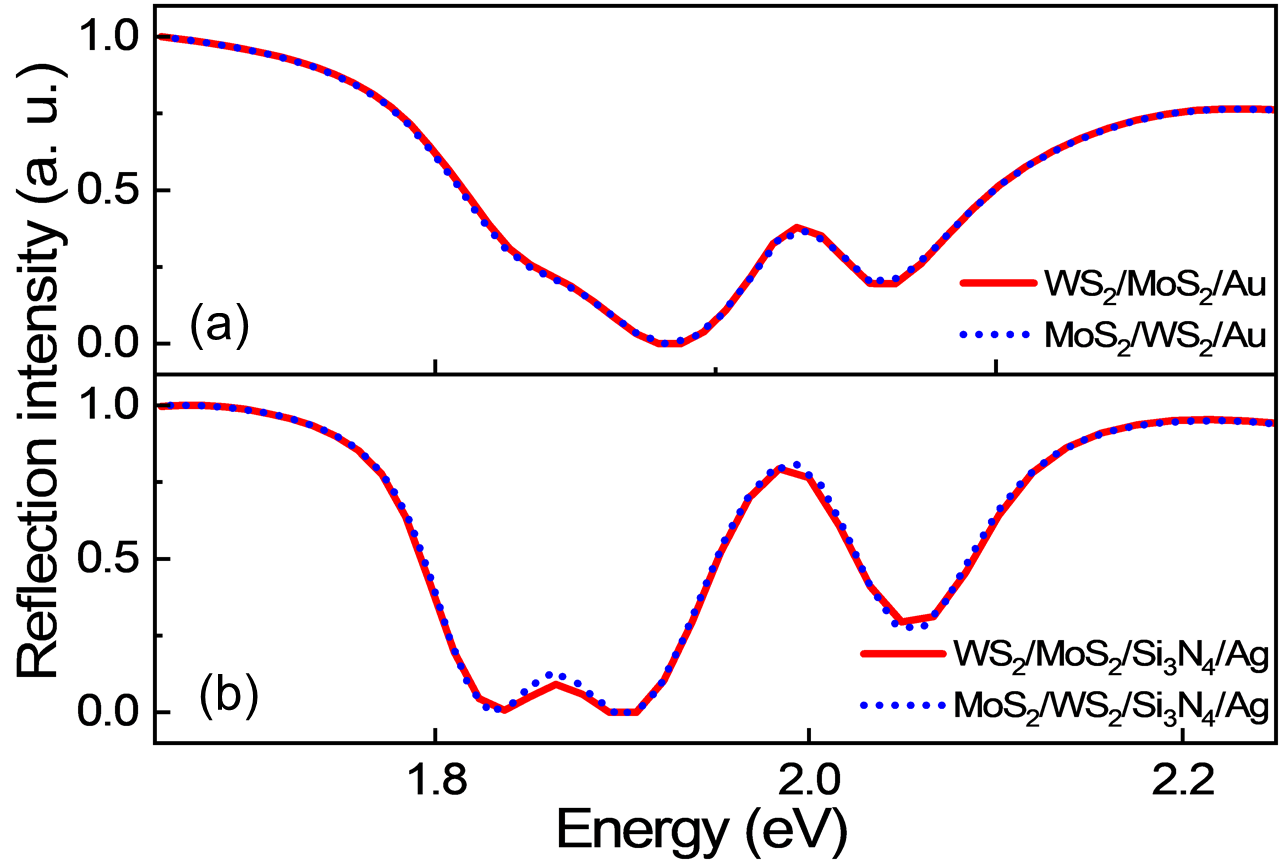


**Figure S1**: (a) Reflection spectra calculated for the MoS_2_/WS_2_/Au and WS_2_/MoS_2_/Au excited by using *p*-polarized light. (b) Reflection spectra calculated for the MoS_2_/WS_2_/Si_3_N_4_/Ag and WS_2_/MoS_2_/Si_3_N_4_/Ag structure excited by using *s*-polarized light.

Supplementary Note 2

In Figure S2a, b, we present the electric field distributions (*E*_x_) calculated for the SPPs generated in the MoS_2_/WS_2_/Au structure at the energies of X_A1_ and X_A2_. For comparison, the electric field distributions (*E*_x_) calculated for the SPPs generated in the Au film at the energies of the SPPs are shown in Figure S2c, d. One can see that the amplitude of the electric field is reduced in the presence of the MoS_2_/WS_2_ heterobilayer, indicating the coupling between the SPPs and the excitons. Notably, the resonant energy of the SPP is redshifted in presence of the MoS_2_/WS_2_ due to the screening effect [[1](#_ENREF_1)].


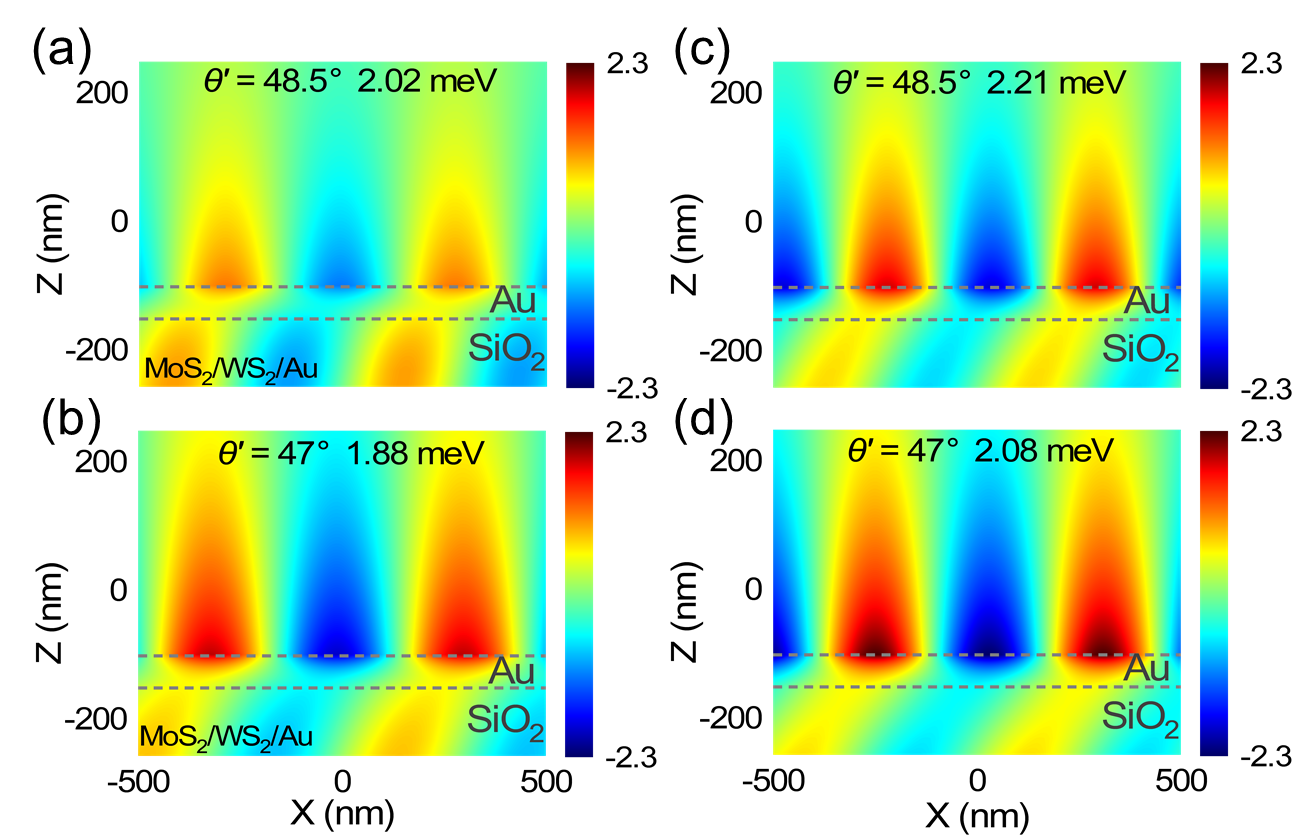


Figure S2. (a)(b) Electric field distributions (*E*_x_) calculated for the SPPs generated in the MoS_2_/WS_2_/Au structure at the energies of X_A1_ and X_A2_. (c)(d) Electric field distributions (*E*_x_) calculated for the SPPs in the Au film at the energies of the SPPs.

**Supplementary Note 3**

In Figure S3a, b, we present the electric field distributions (*E*_y_) calculated for the TE waves excited in the WS_2_/MoS_2_/Si_3_N_4_/Ag structure at the energies of X_A1_ and X_A2_. For comparison, the electric field distributions (*E*_y_) of the TE waves excited in the Si_3_N_4_/Ag at the energies of the TE waves are shown in Figure S3c, d. It can be seen that the amplitude of the electric field in the WS_2_/MoS_2_/Si_3_N_4_/Ag structure is reduced by approximately an order of magnitude as compared with that in the Si_3_N_4_/Ag structure, indicating the energy exchange between the TE wave and the excitons.

**
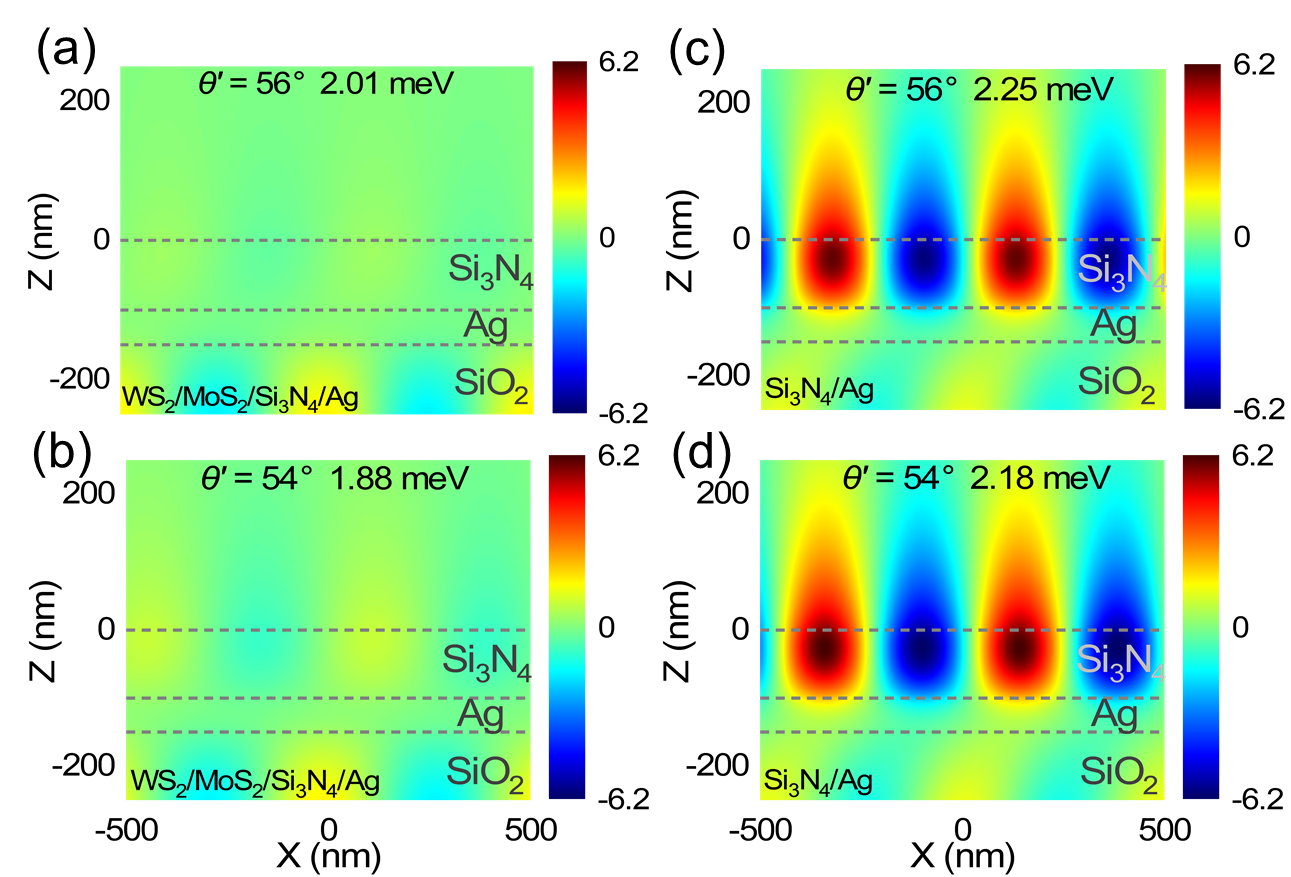
**

Figure S3. (a)(b) Electric field distributions (*E*_y_) calculated for the TE waves excited in the WS_2_/MoS_2_/Si_3_N_4_/Ag structure at the energies of X_A1_ and X_A2_. (c)(d) Electric field distributions (*E*_y_) calculated for the TE waves excited in the Si_3_N_4_/Ag at the energies of the TE waves.

**Supplementary Note 4**

Basically, the photon-exciton coupling strength can be expressed as follows:

. (1)

Here, *V* is the mode volume of the surface wave, which can be written as:

$V=\frac{\int\varepsilon(\vec{r})\left|\vec{E}(\vec{r})\right|^{2}d^{3}\vec{r}}{\max(\varepsilon(\vec{r})\left|\vec{E}(\vec{r})\right|^{2})}$. (2)

Actually, the coupling between the plasmonic mode supported by a periodic array of metallic nanoparticles and the exciton resonance of a two-dimensional material has been widely studied [[2](#_ENREF_2), [3](#_ENREF_3)]. In addition, the coupling between the optical mode supported by a dielectric metasurface and the exciton resonance of a two-dimensional material was also investigated [[4](#_ENREF_4), [5](#_ENREF_5)]. In these cases, the mode volume of the plasmonic (or optical) mode, which belongs to a nonlocal mode, was evaluated by considering one unit cell of the periodic structure [[6](#_ENREF_6), [7](#_ENREF_7)]. In Figure S4, we present the electric field distributions in the XZ and YZ planes calculated for the SPP excited on the Au film and these for the TE wave excited on the Si_3_N_4_/Ag structure at the energy of X_A1_. Similarly, we can consider a region with one wavelength in both the X and Y directions when we calculate the mode volumes of the surface waves. In this way, the mode volumes of the SPP and TE waves are derived to be ~4.29×10^-2^ and ~2.73×10^-2^ μm^3^, respectively. These values are moderate as compared with those reported in previous studies [[8](#_ENREF_8), [9](#_ENREF_9)]. It should be emphasized that the coupling strength is not only inversely proportional to the square root of the mode volume (*V*) but also proportional to the square root of the number of excitons involved in the coupling (*N*). Although the surface waves studied in this work possess relatively large mode volumes, the number of excitons involved in the coupling is also large. This is the reason why strong coupling can be easily achieved between the TE waves and two-dimensional materials. Therefore, the smaller mode volume of the TE wave is beneficial for realizing strong coupling with the three excitons in the heterobilayer.


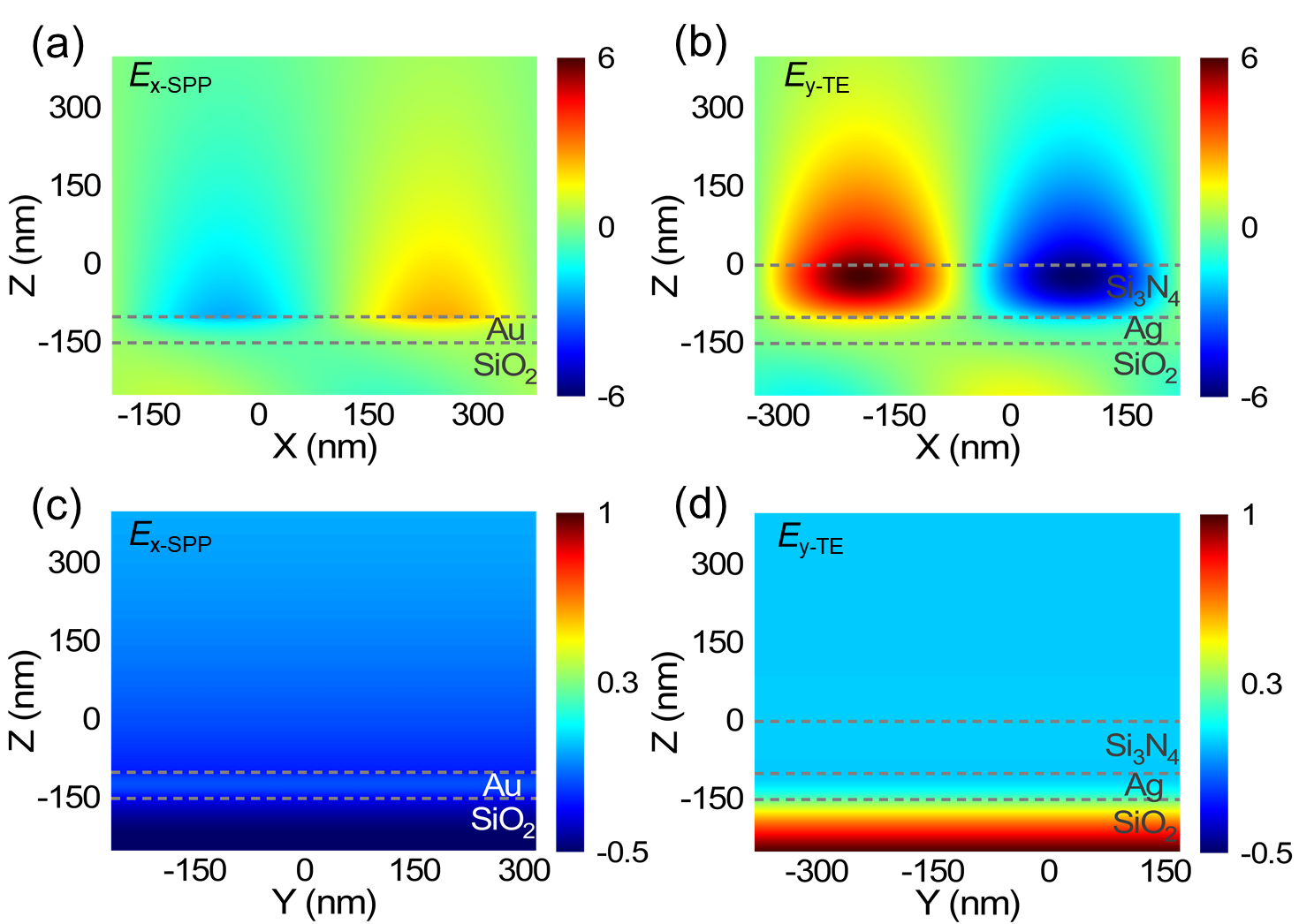


**Figure S4**: (a)(c) Electric field distributions (*E*_x_) in the XZ and YZ planes calculated for the SPP excited on the Au film at the energy of X_A1_. (b)(d) Electric field distributions (*E*_y_) in the XZ and YZ planes calculated for the TE wave excited on the Si_3_N_4_/Ag structure at the energy of X_A1_.

**Supplementary Note 5**

In Figure S5, we present the calculated reflection spectra of the SPP and TE wave excited by using *p*- and *s*-polarized light, respectively. One can see that the linewidth of the TE wave is much narrower than that of the SPP. The linewidths of the SPP and TE wave are estimated to be ~190.85 and ~27.84 meV based on the reflection spectra.

We analyzed the influence of the linewidth of the surface wave on the Rabi splitting energy in a coupled system. The hybrid states formed by the coupling between a photon and an exciton can be described by using a 2×2 Hamiltonian, which is describes as follows [[10](#_ENREF_10)]

$\left( \begin{matrix} E_{c}-i\frac{\gamma_{c}}{2} & g \\ g & E_{x}-i\frac{\gamma_{x}}{2} \end{matrix} \right)\left( \begin{matrix} \alpha\\ \beta\end{matrix} \right)=E_{\pm}\left( \begin{matrix} \alpha\\ \beta\end{matrix} \right),$ (3)

where *E_j_* (*j* = c, x) represent the uncoupled energies of the optical mode and the exciton, respectively; γ*_j_* (*j* = c, x) denote the dissipation rates (linewidths) of the optical mode and exciton, respectively; *α*, *β* are the Hopfield coefficients of the two hybrid states; and *E_±_*​ are the eigenenergies of the two hybrid states. The eigenenergies of the two hybrid states are expressed as follows:

$E_{\pm}=\frac{E_{c}+E_{x}}{2}-i\frac{\gamma_{c}+\gamma_{x}}{4}\pm\sqrt{g^{2}+\frac{1}{4}\left[ \delta-\frac{1}{2}i\left( \gamma_{c}-\gamma_{x} \right) \right]^{2}} ,$ (4)

where $\delta=E_{c}-E_{x}$ denotes the detuning. When $E_{c}=E_{x}$​, $\delta=0$. At this point, the eigenenergies are written as:

$E_{\pm}=E_{j}-i\frac{\gamma_{c}+\gamma_{x}}{4}\pm\sqrt{g^{2}-\frac{1}{16}{(\gamma_{c}-\gamma_{x})}^{2}}$ . (5)

The Rabi splitting energy *Ω* representing the energy difference between the upper branch and the lower one is given by:

$\Omega=E_{+}-E_{-}=2\sqrt{g^{2}-\frac{1}{16}{(\gamma_{c}-\gamma_{x})}^{2}}$ . (6)

In the main text, we have provided the coupling strengths between the two surface waves and the three excitons, as well as the linewidths of the three excitons. Substituting into eq. 6, we can obtain the Rabi splitting energies of the coupling between the two surface waves and the three excitons, which are summarized in the Table 1. It can be seen that the Rabi splitting energies originating from the coupling between the TE waves and the three excitons are larger than those originating from the SPPs and three excitons. These results are in good agreement with those observed in the calculated reflection spectra and measured scattering spectra.


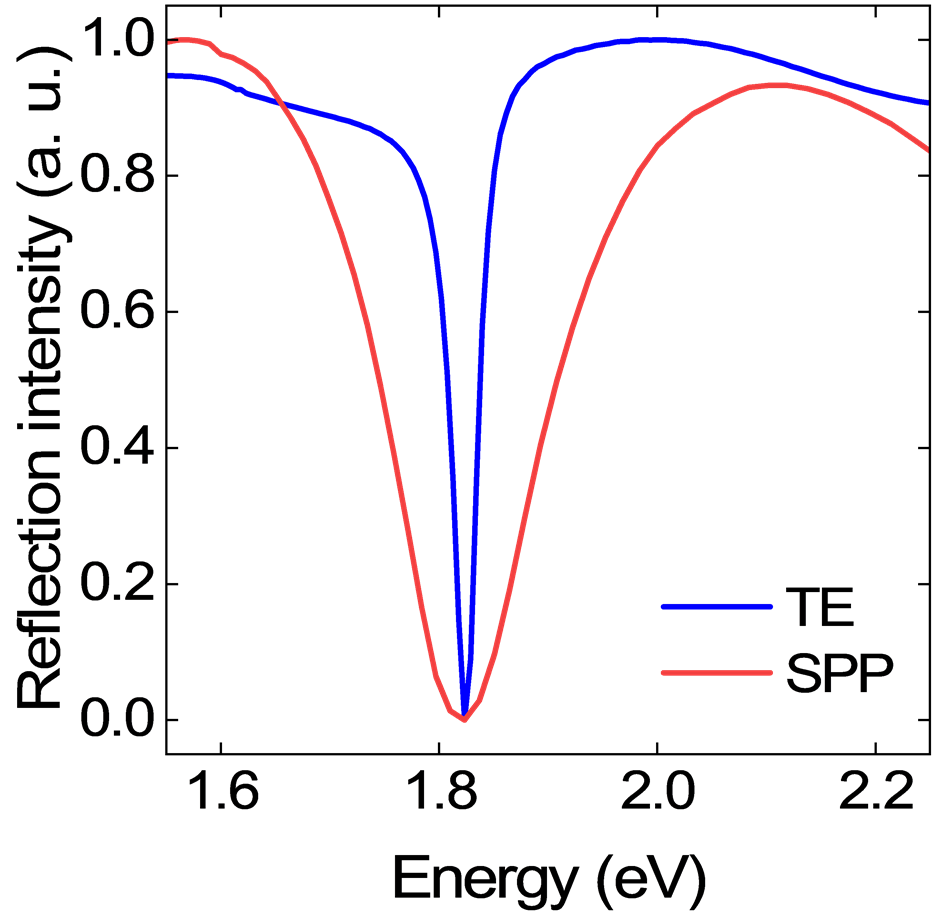


**Figure S5.** Calculated reflection spectra of the SPP and TE wave.

**Table 1** Rabi splitting energies observed in the coupling between the surface waves (SPP and TE wave) and the three excitons.

| Ω (meV) | X_A1_ | X_B_ | X_A2_ |
| --- | --- | --- | --- |
| SPP | 100.71 | 64.79 | 93.03 |
| TE | 113.97 | 83.52 | 127.28 |

**Supplementary Note 6**

In Figure S6, we present the two-dimensional reflection spectra calculated for the Au film, WS_2_/Au, and MoS_2_/WS_2_/Au structures excited by using *p*-polarized light with different incident angles ($\theta^{'}$). In Figure S6a, it can be seen that the resonant energy of the SPP is blueshifted with increasing incident angle. In Figure S6b, an anti-crossing behavior is observed at the energy of X_A1_, indicating the coupling between the SPP and X_A1_. The dispersion relations of the two hybrid states are represented by the blue and red curves. In Figure S6c, the anti-crossing behaviors expected at the energies of X_A2_ and X_B_ are not apparent.


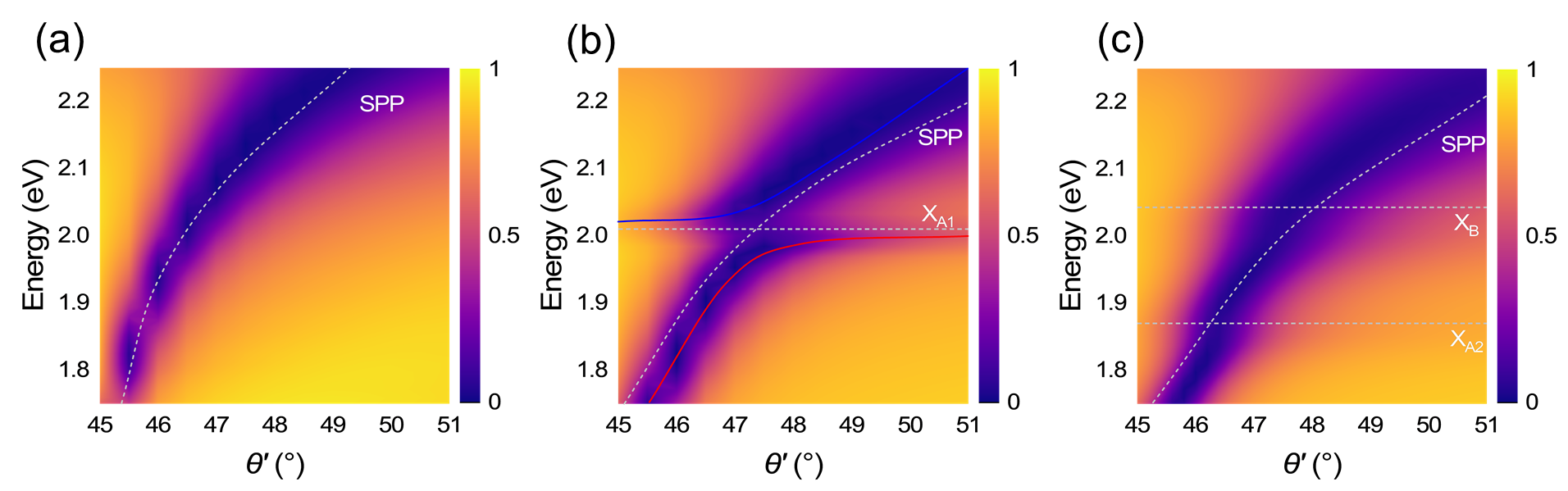


**Figure S6.** Angle-resolved two-dimensional reflection spectra calculated for the Au film (a), WS_2_/Au (b), and MoS_2_/WS_2_/Au (c) structures. The energies of the SPP and the three excitons are indicted by dashed curves and lines, respectively.

**Supplementary Note 7**

In Figure S7, we present the two-dimensional reflection spectra calculated for the Si_3_N_4_/Ag, WS_2_/Si_3_N_4_/Ag, and WS_2_/MoS_2_/Si_3_N_4_/Ag structures excited by using *s*-polarized light with different incident angles. In Figure S7a, it can be seen that the resonant energy of the TE wave is blueshifted with increasing incident angle. In Figure S7b, one can see an anti-crossing behavior at the energy of X_A1_, leading to the formation of two hybrid states (marked by blue and red curves). In Figure S7c, one can identify pronounced anti-crossing behaviors at the energies of X_A1_ and X_A2_, resulting in upper, middle, and lower branches (marked by blue, green, and red curves).


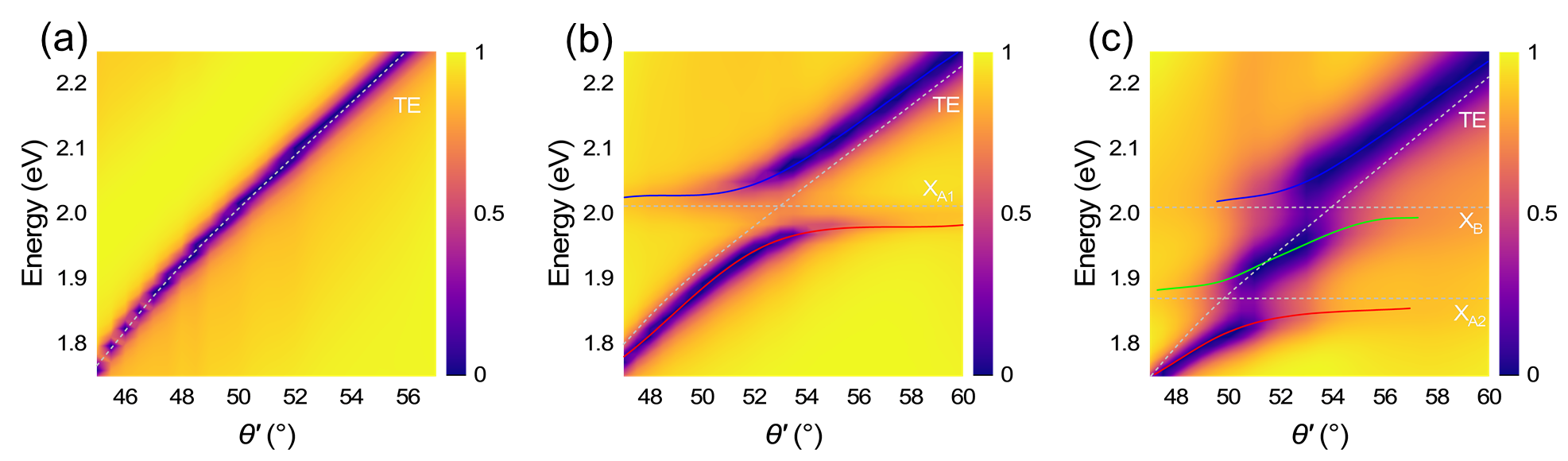


**Figure S7.** Angle-resolved two-dimensional reflection spectra calculated for the Si_3_N_4_/Ag (a), WS_2_/Si_3_N_4_/Ag (b), and WS_2_/MoS_2_/Si_3_N_4_/Ag (c) structures. The energies of the TE waves and the three excitons are indicated by dashed curves and lines, respectively.

**Supplementary Note 8**

In Figure S8a, b, we present the forward scattering spectra of a PS nanosphere placed on a Al_2_O_3_ substrate and an oligomer of PS nanospheres located on a Si_3_N_4_/Ag structure. In both cases, the scattering spectra appear as the broadbands without any optical resonances in the visible light spectrum. In Figure S8c, the two dips observed in the scattering spectrum of an oligomer of PS nanospheres located on the WS_2_/MoS_2_/Si_3_N_4_/Ag structure arise from the optical absorption of the A-excitons in the WS_2_ and MoS_2_ monolayer, respectively. Thus, the use of an oligomer of PS nanospheres as the scatter for the surface wave does not affect the coupling between the surface wave and the excitons.


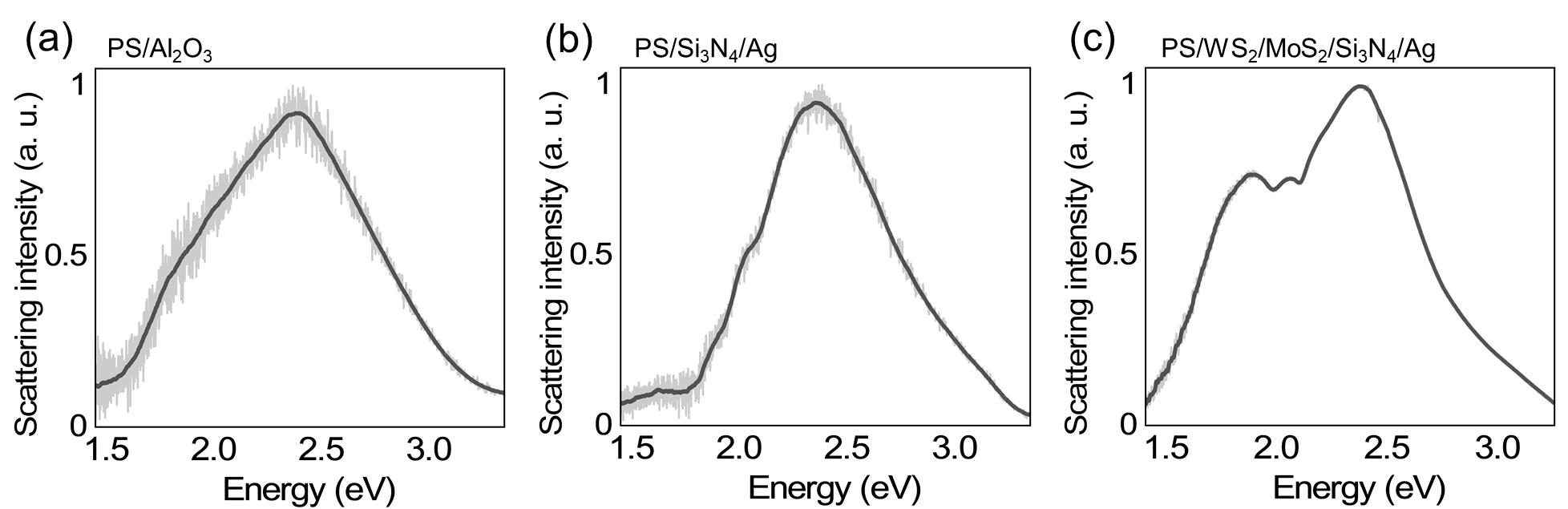


**Figure S8.** Forward scattering spectra measured for the PS/Al_2_O_3_ (a), PS/Si_3_N_4_/Ag (b) and PS/WS_2_/MoS_2_/Si_3_N_4_/Ag (c), respectively.

**Supplementary Note 9**

In Figure S9, we present the angle-resolved scattering spectra of the oligomers of PS nanospheres located on a MoS_2_/WS_2_/Au and a WS_2_/MoS_2_/Si_3_N_4_/Ag structure, respectively. Similar to the phenomenon observed in Figure 9, one can identify two dips and three peaks in the scattering spectra. In addition, it is apparent that the dips observed in Figure S9b are deeper than those observed in Figure S9a, indicating stronger coupling between the TE wave and the three excitons as compared with that between the SPP and the three excitons.


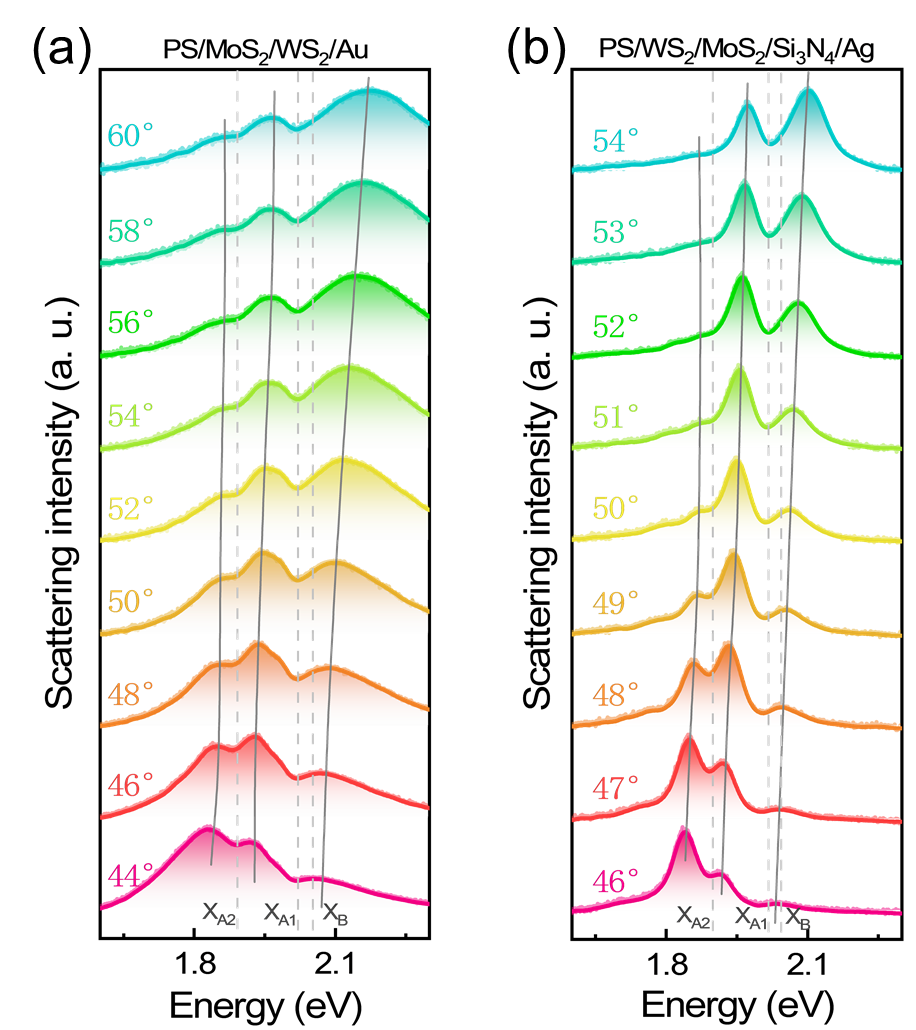


**Figure S9.** Angle-resolved scattering spectra measured for the oligomers of PS nanospheres located on a MoS_2_/WS_2_/Au (a) and a WS_2_/MoS_2_/Si_3_N_4_/Ag structure (b). The dashed lines indicate the energies of the three excitons while the solid lines indicate the energies of the three hybrid states.

**Supplementary Note 10**

In Figure S10a, b, we present the angle-resolved scattering spectra of the oligomers of PS nanospheres located on a WS_2_/Au and a MoS_2_/Au structure. In Figure S10a, one can see a dip and two peaks in the scattering spectra originating from the coupling between the SPP and X_A1_. Figure S10c show the dispersion relations of the two hybrid states fitted by using a 2×2 Hamiltonian. The derived coupling strength (*g* = 79 meV) exceeds the averaged dissipation rate ((*γ_cav_* + *γ_ex_*)/4), satisfying the strong coupling criterion [[11](#_ENREF_11)].

In Figure S10b, two shallow dips and three peaks are observed at the energies of X_A2_ and X_B_ in scattering spectra. In Figure S10d, we present the dispersion relations of the three hybrid states fitted by using a 3×3 Hamiltonian and derived the coupling strengths (*g*_SPP-X_*_i_ _(i = A2, B)_* = 70, 75 meV). Due to the large dissipation rate of X_B_ (132 meV), the strong coupling criterion is not fulfilled, indicating that the strong coupling between the SPP and the two excitons in the MoS_2_ monolayer is not achieved.


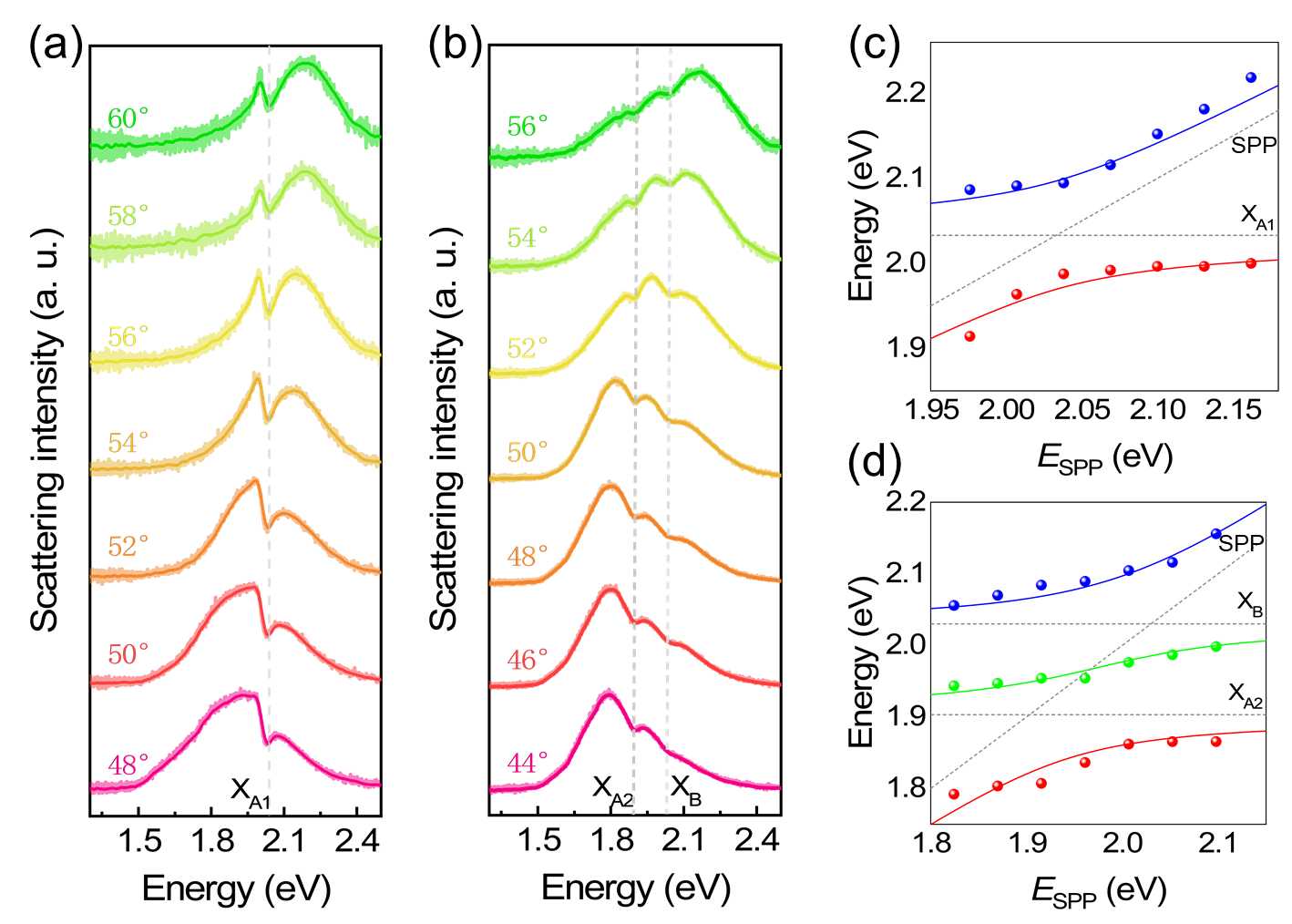


**Figure S10.** (a) Scattering spectra measured for an oligomer of PS nanospheres placed on a WS_2_/Au (a) and a MoS_2_/Au structure (b) at different incident angles. The energies of the three excitons are indicated by the dashed lines. (c)(d) Dispersion relations of the hybrid states extracted from the scattering spectra (solid symbols) shown in (a) and (b). Also shown are the fitting results of the dispersion relations based on the Hamiltonian (solid curves). The energies of the SPP and the excitons are indicated by dashed lines.

**Supplementary Note 11**

Figure S11a shows the bright-field image of a MoS_2_/WS_2_ heterostructure attached on a thin Au film. It consists of a few-layer WS_2_ (green area) and a few-layer MoS_2_ (blue area) obtained by using the mechanical exfoliation technique. Figure S11b shows the Raman spectra measured for the few-layer WS_2_, MoS_2_, and MoS_2_/WS_2_. The four peaks indicate that the heterobilayer is composed of few-layer WS_2_ and MoS_2_. In Figure S11c, we present the angle-resolved scattering spectra measured for an oligomer of PS nanospheres located on the MoS_2_/WS_2_ heterostructure. One can see the two dips and three peaks in the scattering spectra, similar to the phenomenon observed in Figure 4a. It is noteworthy that the dips at lower energies are clearer as compared with those observed in the MoS_2_/WS_2_ heterobilayer on the Au film because more excitons in the MoS_2_/WS_2_ heterostructure are involved in the coupling.

In Figure S11d, we present the dispersion relations of the hybrid states extracted from the scattering spectra shown in Figure S11c (solid symbols). The fitting results based on the Hamiltonian are represented by the solid curves. We obtained a Rabi splitting energy of ~261.20 meV, which is higher than that observed in the MoS_2_/WS_2_ heterobilayer. In addition, the coupling strengths *g*_SPP-X_*_i_ _(i = A1, A2, B)_* = 88, 57,50 meV between the SPP and the three excitons are also larger than those observed in the MoS_2_/WS_2_ heterobilayer. However, the strong coupling criterion is still not satisfied for the coupling between the SPP and X_A2_ (X_B_).


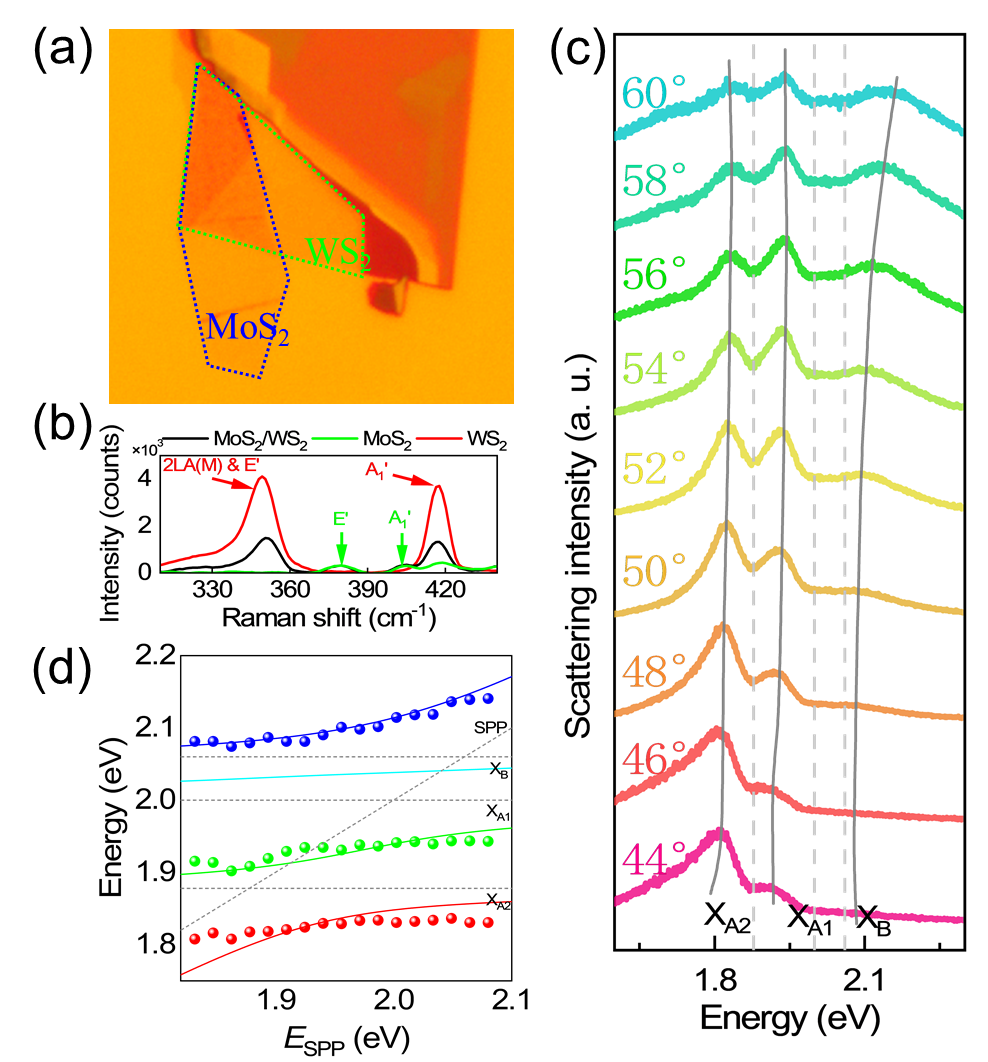


**Figure S11.** (a) Bright-field image of a MoS_2_/WS_2_ heterostructure composed of a few-layer WS_2_ (green area) and a few-layer MoS_2_ (blue area) attached on a thin Au film. (b) Raman scattering spectra measured for the MoS_2_/WS_2_ heterostructure (black curve) and the few-layer WS_2_ (red curve) and MoS_2_ (green curve). (c) Scattering spectra measured for an oligomer of PS nanospheres placed on the MoS_2_/WS_2_ heterostructure at different incident angles. The three vertical dashed lines indicate the energies of the three excitons while the three gray solid lines are indicated the three hybrid states. (d) Dispersion relations of the four hybrid states extracted from the scattering spectra (solid symbols). Also shown are the fitting results of the dispersion relations based on the Hamiltonian (solid curves). The energies of the SPP and the three excitons are indicated by dashed lines.

**Supplementary Note 12**

In Figure S12a, b, we also present the angle-resolved scattering spectra of the oligomers of PS nanospheres located on a WS_2_/Si_3_N_4_/Ag and a MoS_2_/Si_3_N_4_/Ag structure. In Figure S12a, one can observe a deep dip and two peaks in the scattering spectra originating from the coupling between the TE wave and X_A1_. In Figure S12c, we present the dispersion relations of the two hybrid states fitted by using a 2×2 Hamiltonian. The derived coupling strength (*g* = 50 meV) exceeds the averaged dissipation rate (*γ_cav_* + *γ_ex_*)/4, meeting the strong coupling criterion [[12](#_ENREF_12)].

In Figure S12b, two shallow dips and three peaks are observed at the energies of X_A2_ and X_B_ in the scattering spectra. In Figure S12d, we present the dispersion relations of the three hybrid states fitted by using a 3×3 Hamiltonian and derived the coupling strengths (*g*_TE-X_*_i_ _(i = A2, B)_* = 53, 40 meV). The strong coupling criterion is satisfied, indicating the achievement of the strong coupling between the TE wave and the two excitons in the MoS_2_ monolayer.


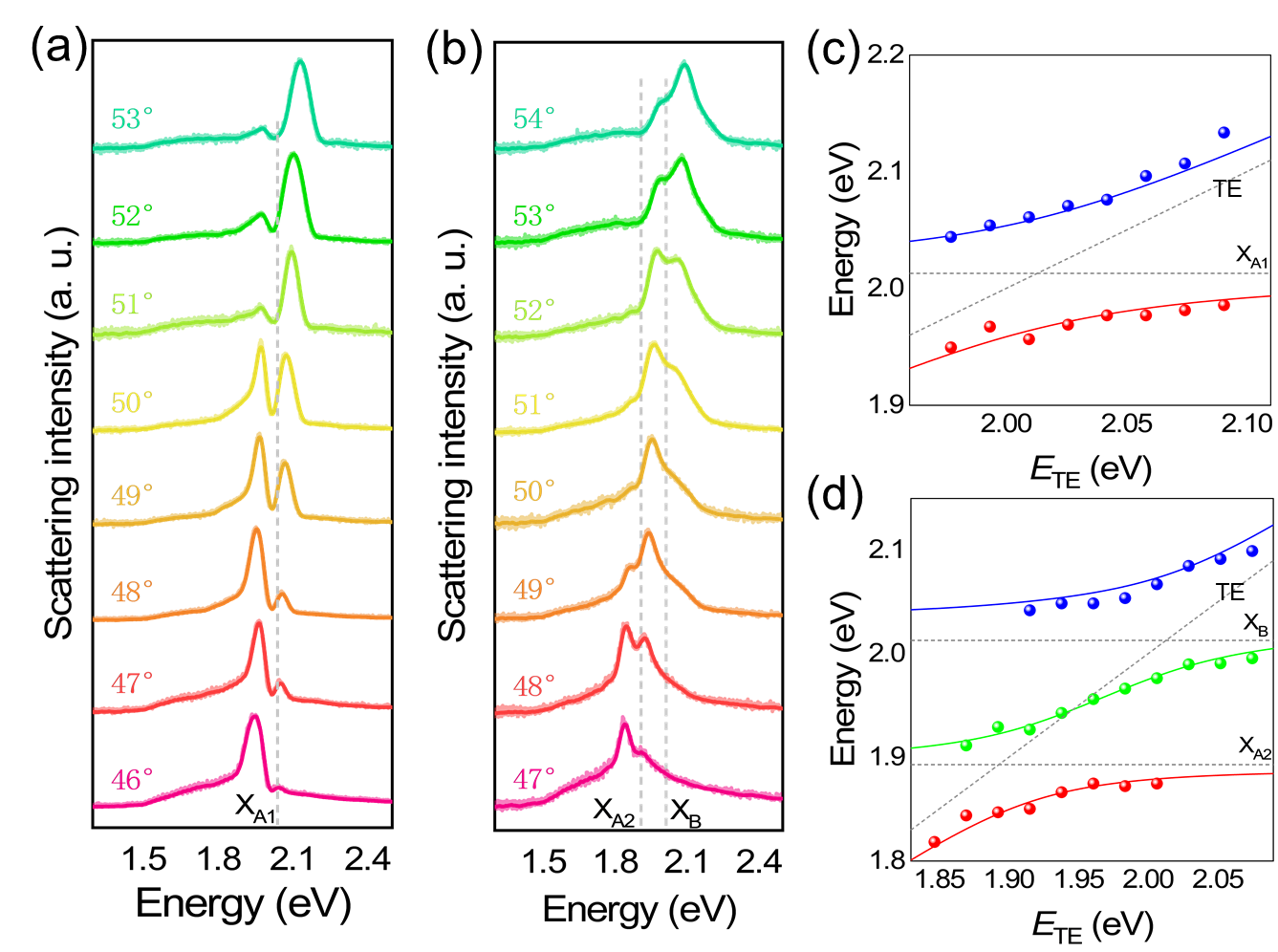


**Figure S12.** (a) Scattering spectra measured for an oligomer of PS nanospheres placed on a WS_2_/Si_3_N_4_/Ag (a) and a MoS_2_/Si_3_N_4_/Ag structure (b) at different incident angles. The energies of the three excitons are indicated by the dashed lines. (c)(d) Dispersion relations of the hybrid states extracted from the scattering spectra (solid symbols) shown in (a) and (b). Also shown are the fitting results of the dispersion relations based on the Hamiltonian (solid curves). The energies of the TE wave and the excitons are indicated by dashed lines

**Supplementary Note 13**

In Figure S13, we present the current density distributions calculated in a Si_3_N_4_/Ag structure at resonances of the TE wave (at two incident angles). In these cases, the maximum current density is located in the Si_3_N_4_ layer_._

**
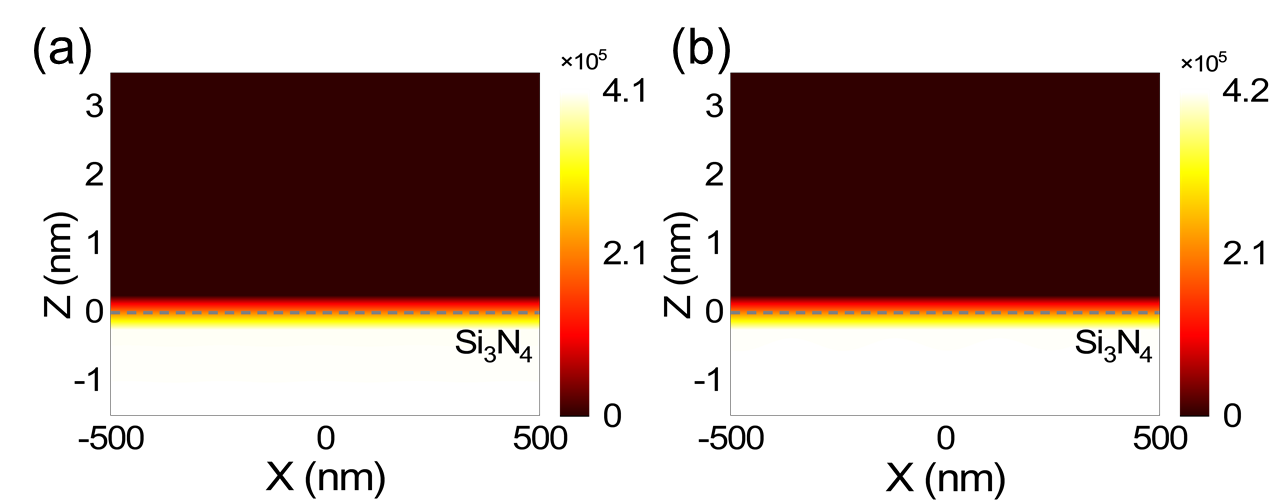
**

**Figure S13.** (a) Current density distribution calculated for a Si_3_N_4_/Ag structure at the resonant energy of the TE wave (2.25 eV) excited at an incident angle of 56°. (b) Current density distribution calculated for a Si_3_N_4_/Ag structure at the resonant energy of the TE wave (2.18 eV) excited at an incident angle of 54°.

**References**

[1] A. Q. Hu, W. D. Zhang, S. Liu, et al., ''In situ scattering of single gold nanorod coupling with monolayer transition metal dichalcogenides,'' *Nanoscale*, vol. 11, no. 43, pp. 20734–20740, 2019.

[2] L. Liu, L. Y. M. Tobing, X. Yu, et al., ''Strong plasmon-exciton interactions on nanoantenna array-monolayer WS_2_ hybrid system,'' *Adv. Opt. Mater.*, vol. 8, no. 5, 2019.

[3] S. Wang, Q. Le-Van, F. Vaianella, et al., ''Limits to strong coupling of excitons in multilayer WS_2_ with collective plasmonic resonances,'' *ACS Photonics*, vol. 6, no. 2, pp. 286–293, 2019.

[4] I. A. M. Al‐Ani, K. As'Ham, L. Huang, A. E. Miroshnichenko,H. T. Hattori, ''Enhanced strong coupling of TMDC monolayers by bound state in the continuum,'' *Laser Photon. Rev.*, vol. 15, no. 12, 2021.

[5] S. Cao, H. Dong, J. He, et al., ''Normal-incidence-excited strong coupling between excitons and symmetry-protected quasi-bound states in the continuum in silicon nitride-WS_2_ heterostructures at room temperature,'' *J. Phys. Chem. Lett.*, vol. 11, no. 12, pp. 4631–4638, 2020.

[6] Y. Liang, D. P. Tsai,Y. Kivshar, ''From local to nonlocal high-Q plasmonic metasurfaces,'' *Phys. Rev. Lett.*, vol. 133, no. 5, 2024.

[7] Y. Liang, K. Koshelev, F. Zhang, et al., ''Bound states in the continuum in anisotropic plasmonic metasurfaces,'' *Nano Lett.*, vol. 20, no. 9, pp. 6351–6356, 2020.

[8] D. G. Baranov, M. Wersall, J. Cuadra, T. J. Antosiewicz,T. Shegai, ''Novel nanostructures and materials for strong light matter interactions,'' *ACS Photonics*, vol. 5, no. 1, pp. 24–42, 2018.

[9] G. Zengin, M. Wersäll, S. Nilsson, et al., ''Realizing strong light-matter interactions between single-nanoparticle plasmons and molecular excitons at ambient conditions,'' *Phys. Rev. Lett.*, vol. 114, no. 15, p. 6, 2015.

[10] P. Törmä,W. L. Barnes, ''Strong coupling between surface plasmon polaritons and emitters: A review,'' *Rep. Prog. Phys.*, vol. 78, no. 1, p. 34, 2015.

[11] F. Deng, H. F. Liu, L. Xu, S. Lan,A. E. Miroshnichenko, ''Strong exciton-plasmon coupling in a WS_2_ monolayer on Au film hybrid structures mediated by liquid ga nanoparticles,'' *Laser Photon. Rev.*, vol. 14, no. 4, p. 8, 2020.

[12] S. L. Li, L. D. Zhou, F. Deng, et al., ''Transverse-electric-polarized polaritons propagating in a WS_2_/Si_3_N_4_/Ag heterostructure,'' *Laser Photon. Rev.*, vol. 16, no. 12, p. 9, 2022.
